# Packing, Flow and Aerosolization Properties of Binary Adhesive Mixtures Containing Micronized and Spray-Dried Drugs

**DOI:** 10.3390/pharmaceutics18091092

**Published:** 2026-08-30

**Authors:** Anna Simonsson, Nicklas Bunta Sundin, Tobias Bramer, Alex Wimbush, Göran Alderborn

**Affiliations:** 1 Department of Pharmaceutical Biosciences and the Swedish Drug Delivery Center, Uppsala University, P.O. Box 591, SE-751 24 Uppsala, Sweden; annasimonsson4@gmail.com (A.S.); nicklas.sundin@uu.se (N.B.S.); 2 Global Product Development, Pharmaceutical Technology & Development, AstraZeneca Gothenburg, SE-431 53 Mölndal, Sweden; tobias.bramer@astrazeneca.com; 3 Sustainable Innovation and Transformational Excellence, Pharmaceutical Technology & Development, AstraZeneca Gothenburg, SE-431 53 Mölndal, Sweden; alex.wimbush@astrazeneca.com

**Keywords:** binary adhesive mixture, inhalation, drug solid form, powder mechanical properties, mixture dispersion properties

## Abstract

**Background/Objectives:** Packing, flow, and aerosolization properties of a series of binary adhesive mixtures containing micronized or spray-dried drugs were investigated, and the relationships between these blend properties and the blend structure were studied. **Methods:** Micronized or spray-dried terbutaline sulfate and salbutamol sulfate were used as model drugs, and an α-lactose powder was used as the carrier. Binary mixtures with drug loads ranging from 2 to 20% were prepared. The bulk density, compressibility, permeability, and shearing properties of the carrier powder and mixtures were determined, along with the in vitro aerosolization propensity of the mixtures using two types of inhalers. Imaging of the mixtures was used to assess the blend structure. **Conclusions:** The particle engineering method gave differences in particle crystallinity and morphology. The development of the adhesive layer with drug load was broadly consistent with the blend state concept. Spray-dried particles, however, exhibited a higher propensity to localize within surface cavities on the carrier and produced a more voluminous enveloped adhesive layer. The spray-dried particles gave a higher bulk density, a lower Hausner ratio, comparable shear strength, and a lower angle of internal friction. Aerosolization performance, including metrics such as fine particle fraction (*FPF*), depended on inhaler design; nevertheless, for both inhalers, aerosolization behavior was influenced by blend state and physicochemical properties of the drug. At low drug loads, spray-dried particles dispersed to a lower degree, while at high drug loads, the dispersion performance of the two particle types converged. For example, at an intermediate drug load of 7.4%, the *FPF* was about 20% for the spray-dried drugs and about 30% for the micronized drugs using the Screenhaler device, while the corresponding *FPF*:*s* were about 25% and 50% for spray-dried drugs and 40% and 55% for crystalline drugs using the Monodose inhaler. Overall, the physical characteristics of the drug particles were found to influence the structural evolution of the blends, as well as their mechanical and aerosolization properties.

## 1. Introduction

Aerosol drug delivery to the lungs via inhalation is used for different medical purposes [1,2]. The combination of formulation and aerosolization device is crucial for ensuring reproducible and effective drug delivery, i.e., the delivery of particles with an appropriate aerodynamic diameter that can reach the intended target areas of the respiratory tract. Commonly used devices include nebulizers, pressurized metered-dose inhalers (pMDIs), and dry powder inhalers (DPI) [3]. In a DPI, the type of powder in the formulation may vary; one of these is adhesive mixtures, consisting of an assembly of adhesive units in which fine drug particles are adhered to a carrier [4]. In such formulations, a third agent, typically fine lactose or magnesium stearate, may also be used to improve drug aerosolization [5]. Adhesive mixtures represent a formulation approach that provides good powder packing and flow properties combined with an acceptable degree of drug dispersion [6,7,8].

Drug particles adhered to a carrier surface can be organized in different ways depending on the drug concentration, i.e., the adhesive layer shows different architecture depending on the drug load [9]. To describe, in a simplified way, the changes in the physical state of the mixture that occur as drug load increases, Rudén et al. [10] introduced the concept of blend state. This concept is based on the assumption that changes in certain powder properties (such as bulk density or Hausner ratio) reflect changes in the architecture of the adhesive layer and the existence of free self-agglomerates of the fine drug. This structural evolution was later summarized in five blend states [11], ranging from “S1”, in which drug particles are deposited mainly in carrier cavities, to “S3”, in which the adhesive layer has thickened to such an extent that drug particles can no longer attach to the carrier surface and instead form self-agglomerates. Accordingly, adhesive mixtures can be described using two saturation levels: the first occurs when the carrier’s surface cavities are filled with fine drug powder (carrier cavity saturation), and the second when the maximum carrying capacity of the carrier is reached (total carrier saturation).

Adhesive mixtures are typically used at low to intermediate drug loads [12]. However, the drug concentration can nevertheless vary significantly, approximately 0.05–10% [13], potentially leading to large variations in the structure of the adhesive layer. The degree of drug dispersion, often assessed using the fine particle fraction (*FPF*) calculated from in vitro impactor experiments, can vary considerably depending on the type of DPI and the specific formulation used [14,15,16,17]. One factor contributing to such variations may be the physical state of the adhesive mixture, including the structure of the adhesion layer and the strength of the particle-particle interactions within the adhesion units.

In some earlier papers, potential links between the structure of adhesive mixtures for inhalation (blend state) and their performance have been studied. Sarangi et al. [18] proposed that the detachment of drug particles from adhesive units during vibration (i.e., blend mechanical stability or segregation) occurs most easily from flat carrier surfaces (i.e., blend state 2), while particles adsorbed within surface cavities (blend state 1) are more difficult to detach. Simonsson et al. [11] showed that the dispersibility of adhesive blends for one combination of carrier and drug (budesonide) varied in an intricate way with drug load, and this was attributed to changes in the spatial distribution of drug particles within the mixture (differences in deposition sites, structure of the adhesive layer and formation of self-agglomerates). Simonsson et al. also reported [19] that, for the same mixtures of carrier and budesonide, the dispersibility correlated with certain shearing and packing properties of the mixtures. The observed variations were suggested to arise from differences in blend architecture and the structure of the adhesive layer.

To summarize, for a given formulation, a link appears to exist between the structure of the adhesive layer and the inhalation powder performance in terms of segregation, packing density, flowability, and aerosolization propensity. In other words, changes in adhesive layer architecture may simultaneously affect all of these properties. Therefore, blend state can be seen as a fundamental mixture characteristic that underlies several derived performance properties. Understanding structure–property relationships for inhalation powders may therefore be critical for optimizing aerosol therapy.

Besides drug concentration and carrier properties, several physical attributes of the drug particles in an adhesive mixture, such as size, shape, crystallinity, and surface morphology, may also affect the structure of the adhesive layer. We now intend to further explore blend state-blend property relationships in binary adhesive mixtures by using drugs with different solid forms.

In recent years, the engineering of inhalable particles has attained increased interest as a means to optimize the performance of inhalation powders, and several engineering approaches are reported [20,21]. Spray drying appears to be a key processing technology in this context, a technology which also can be used to prepare amorphous neat drug particles. Pinto and co-workers [22] have earlier studied adhesive mixtures containing both micronized and spray-dried salbutamol. In these studies, one or two drug concentrations within the range of 1 to 10 wt.% were used. The authors concluded that spray-dried drug particles adhered more loosely to the carrier surfaces and were more prone to forming small agglomerates compared with micronized drug particles. Consequently, micronized drug produced more homogeneous mixtures with better aerosolization performance, as reflected in *FPF*. Regarding the flow properties of the adhesive mixtures, several measurement methods were used. At a low drug load (1%), the physical form of the drug had only a limited effect on flowability. At a high drug load (10%), which generally showed somewhat poorer flowability, the amorphous form generally exhibited lower flowability than the crystalline form. Pinto et al. [22] also reported that the tendency to gain triboelectric charges during mixing differed between spray-dried particles and micronized particles. A similar difference in charging behavior between amorphous and spray-dried salbutamol particles during aerosolization was previously reported by Wong et al. [23].

The physical form of the drug particles may consequently affect the performance of adhesive mixtures. Therefore, powders prepared by micronization and spray drying represent an interesting model system to further our understanding of the evolution of the architecture of adhesive mixtures with drug concentration and how changes in functional mixture properties are linked to changes in blend structure, i.e., if parallel blend structure-blend property relationships exist in adhesive mixtures. The aim of this project was thus to investigate the structure, mechanical properties and aerosolization performance of binary adhesive mixtures containing either micronized or spray-dried drug particles. To this end, mixtures with drug concentrations ranging from 2% to 20% *w*/*w* were prepared using Lactopress as the carrier, and terbutaline sulfate and salbutamol sulfate serving as model drugs. The study will advance the understanding of the role of the physical structure of adhesive mixtures for their performance. Moreover, our earlier research on the evolution of blend states focused predominantly on the role of variations in carrier properties, while this study will provide new knowledge on the possibility of affecting the layer structure and global blend architecture by variations in drug physical properties.

A third component is often used in inhalation powders to enhance the performance of adhesive mixtures [24,25]. However, such performance enhancers were not used in this study in order to isolate the effect of drug physical properties on the relationship between blend structure and performance.

## 2. Materials and Methods

### 2.1. Materials

Terbutaline sulfate (*TS*) and salbutamol sulfate (*SS*) were provided by AstraZeneca (Gothenburg, Sweden). Lactopress (spray-dried) was provided by DFE Pharma (Goch, Germany). LC-grade methanol and acetonitrile (for UPLC analysis) were purchased from Solveco (Rosersberg, Sweden) and VWR International (Fontenay-sous-Bois, France), respectively. Milli-Q water was produced in-house using Purelab Flex equipment (ELGA LabWater, High Wycombe, UK).

### 2.2. Preparation of Drug Particles

Particles of both drugs—*SS* and *TS*—were micronized using a fluidized jet mill (Airfilco, Technoprog 2.5, Södertälje, Sweden) by Magle Chemoswede (Lund, Sweden). Nitrogen was used as the milling gas, with an ejector pressure of 4 bar and a milling pressure of 2 bar. The feed rate was set to 1.5 g/min for *SS* and 1.4 g/min for *TS*. To recrystallize any disordered *TS* material formed during micronization, the micronized *TS* powder was subjected to moist air for 3 days at 86% relative humidity (*RH*). This procedure was not considered necessary for the micronized *SS* powder.

A solution containing 1.96% *w*/*w* of a drug (*SS* or *TS*) in Milli-Q water was spray-dried using a Büchi Mini Spray Dryer B-290 (Büchi, Flawil, Switzerland) equipped with a high-performance cyclone and a Büchi Dehumidifier B-296 (Büchi, Flawil, Switzerland) to maintain a *RH* below 30% in the drier. A silicone tube with an inner diameter of 2.0 mm was used to pump the feed solution. Air was used as the drying gas, with an aspiration rate of 100%, a spray flow rate of 120 mL/h, and an air flow rate of 473 L/h. The *SS* solution was spray-dried at an inlet temperature of 190 °C, resulting in an outlet temperature of 90 °C and an approximate yield of 90%. The *TS* solution was spray-dried at an inlet temperature of 195 °C, giving an outlet temperature of 97 °C and a yield of 88%.

The inlet temperatures of 190–195 °C were determined after pre-trials where several combinations of spray-drying parameters were evaluated. The aims were to match the particle size of the spray-dried material to that of the micronized material, secure amorphous particle formation and maximize process yield. Regarding the yield, high inlet temperatures gave drier, less tacky particle surfaces at the point of collection, reducing wall deposition losses. The measured outlet temperatures in combination with the short particle residence time in the drying chamber (≈1.0 s, calculated from the drying chamber volume and aspirator gas flow rate) meant the cumulative thermal exposure of the drugs was considered acceptable.

Following spray drying, the collected drug powders were stored in a desiccator containing phosphorus pentoxide, maintaining a *RH* of approximately 1% at room temperature, for at least three days (before any subsequent analysis was performed) and thereafter during the course of the experimental work.

Micronized *SS* and *TS* were prepared as a single batch while the spray-dried drugs were each prepared as five independent batches. Particle size was measured in triplicate for each of the five batches (n = 5 batches × 3 replicate measurements), and batch-to-batch reproducibility was confirmed with a relative standard deviation (RSD) of <10% between the independent batches.

### 2.3. Apparent Particle Density and Particle Diameter

The apparent particle density (*ρ*_p_) of all starting materials was measured by Helium pycnometry (AccuPyc 1330, Micrometrics Instruments, Nordcross, GA, USA) using a measurement cylinder with a volume of 11.38 cm^3^. Approximately 50% of the cylinder was filled with powder, and the powder weight was recorded. The particle volume was then determined from five consecutive measurements. Reported values represent the mean and standard deviation of two independent samples.

Volumetric particle size distribution of all starting materials was measured using a Malvern Mastersizer 3000 (Malvern Panalytical, Malvern, UK) in dry mode, equipped with an Aero S Dispersion Unit operating at a dispersion pressure of 2 bar, a feed rate of 20%, and a target obscuration rate between 0.5–3%. The measurements were analyzed in general-purpose mode utilizing Mie scattering theory, with particle refractive indices of 1.566 for *SS* and 1.690 for *TS*, and an absorption index of 0.1 for both. Micronized particles were analyzed as non-spherical, whereas the spray-dried particles were analyzed as spherical, in accordance with their observed shapes (see Section 3). Reported values represent the mean and standard deviation of three independent measurements.

### 2.4. Imaging

Scanning electron microscopy (*SEM*) images were taken using a Zeiss 1530 (Carl Zeiss GmbH, Jena, Germany) microscope, operating at an acceleration voltage of 2.5 kV and using an InLens detector at 5–10 kx magnification. Prior to imaging, the samples were coated with gold/platinum. Light microscope images were also taken using a Zeiss SteREO Discovery.V8 (Carl Zeiss GmBH, Oberkochen, Germany).

### 2.5. Powder X-Ray Diffraction

Powder X-ray diffraction (PXRD) patterns of the drug particles in micronized and spray-dried forms were recorded using a benchtop second-generation D2 Phaser diffractometer (Bruker, Karlsruhe, Germany) equipped with a 1-D LYNXEYE XE-T detector. The diffractometer employed Cu Kα radiation (λ = 1.5418 Å) and was operated at a voltage of 30 kV and a current of 10 mA. Samples were packed into a standard specimen holder (A26D332), with a cavity of 25 mm diameter and 1 mm depth, and the powder surface was leveled using a glass slide. Data were acquired from 10–50° 2θ in continuous-scan mode at 0.1° 2θ s^−1^ (quantified as counts per second (Cps)).

### 2.6. Preparation of Binary Mixtures

For each combination of carrier (Lactopress) and drug (micronized or spray-dried *TS*, or micronized or spray-dried *SS*), a series of binary mixtures with drug concentrations ranging from 1.96–20.0% *w*/*w* and a constant carrier weight of 50 g (Table 1) were prepared by dry mixing in a laboratory Turbula 2F2 mixer (Willy A. Bachofen AG, Muttenz, Switzerland). The mixing procedure followed the method described elsewhere [11].

### 2.7. Packing and Flow Properties of the Drugs and Mixtures

Unconsolidated powder bulk density (*ρ*_b_), unconsolidated powder permeability (*k*), and a series of flowability indicators (Hausner ratio (*HR*), cohesion strength (*τ*_0_), flow function coefficient (*ff*_c_), and angle of internal friction (*φ*) were determined for all drug powders and mixtures. The *ρ*_b_ and the *k* were determined using in-house-built instruments, while the *HR* and the shearing properties were measured using a Freeman FT4 powder rheometer (Freeman Technology, Tewkesbury, UK). For all these measurements, the same experimental procedures as detailed elsewhere were used [19].

In all cases, reported values represent the mean and standard deviation of three independent measurements.

### 2.8. Assessment of In Vitro Aerosolization of Adhesive Mixtures

Aerosolization experiments were performed using a Next Generation Impactor (NGI, Copely Scientific, Nottingham, UK) connected to an inhaler device, either a Screenhaler device equipped with a Turbuhaler mouthpiece [26] or a Monodose RS01 device (Plastiape, Osnago, Italy). The NGI procedure was performed as reported earlier [11] using an airflow rate of 70 L/min (Screenhaler) and 65 L/min (Monodose). HPMC capsules of size 3 were used for the Monodose experiments.

The amount of drug at all stages of the impactor was determined by ultra-performance liquid chromatography (UPLC). The UPLC procedure for quantitative analysis of *SS* was reported earlier [18], and the same procedure and settings were also used for quantitative analysis of *TS*.

As indications of the degree of dispersion (aerosolization) of the mixtures during impactor experiments, the fine particle dose (*FPD*), fine particle fraction (*FPF*) and mass median aerodynamic diameter (*MMAD*) were determined as described earlier [11]. In all cases, reported values represent the mean and standard deviation of three independent measurements.

Since the NGI experiments were time-consuming, mixtures of four drug concentrations were subjected to the impactor study, i.e., 1.96, 7.41, 13.0 and 16.7%. These selected drug loads corresponded approximately to mixtures of the four blend states of the blend state model, i.e., 1, 2a, 2b and 3, as evaluated by imaging of the mixtures and their mechanical properties.

### 2.9. Statistical Analysis

Statistical analysis was performed using Pearson correlation analysis (same procedure as used by Simonsson et al. [19]. Significant linear correlations between variables were assumed when *p* < 0.01 (packing and flow data) and *p* < 0.05 (for packing, flow, and aerosolization data). Cut-off values were set at *p* <0.01 for correlations between packing and flow parameters, and at *p* < 0.1 for correlations between mechanical and aerosolization properties.

For the mechanical properties, data from all mixture concentrations were included in the correlation calculations. For correlations involving both mechanical and aerosolization data, only data from the four mixture concentrations used in the NGI experiments were used. The limited number of mixtures chosen for the aerosolization experiments reduced the statistical power in the statistical analysis, and a cut-off value of *p* < 0.1 was therefore used to study correlations between mechanical properties and aerosolization propensity. Consequently, the observed associations between mechanical and aerosolization properties should be interpreted as indicative trends rather than significant correlations.

## 3. Results

### 3.1. Drug and Carrier Properties

**Figure 1:**
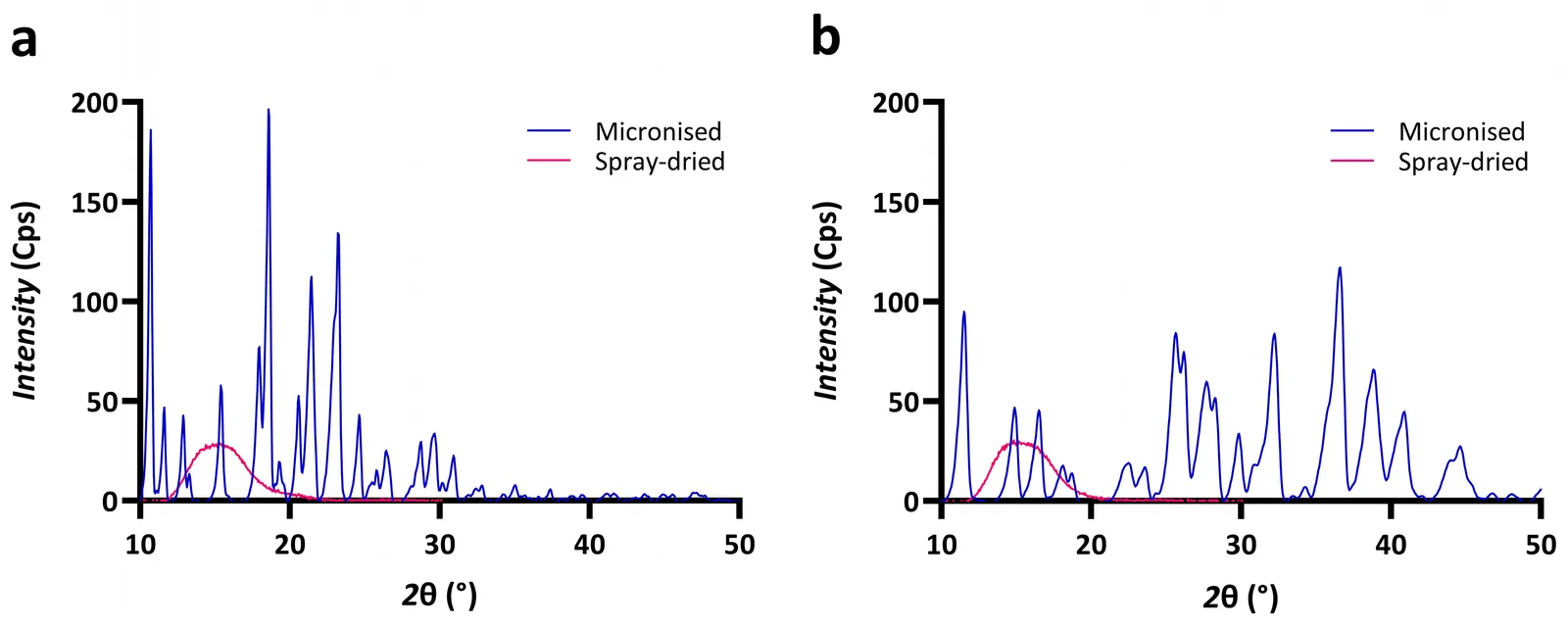
Diffraction patterns of all drug materials. (**a**) SS; (**b**) *TS*.

For the spray-dried drugs, no sharp peaks were observed in the powder X-ray diffraction patterns (Figure 1a,b), indicating a nearly completely disordered state. In contrast, the micronized drugs showed distinct peaks, indicating a predominantly crystalline state. The PXRD of the micronized *TS* gave a diffraction pattern with peaks at 2θ about 11°, 13°, 18°, 21° and 23°. This pattern closely matches the reported diffractogram for terbutaline sulfate anhydrate B, the thermodynamically stable crystal modification [27]. The diffraction pattern of the micronized *SS* particles compared favorably to earlier published data [28].

**Figure 2:**
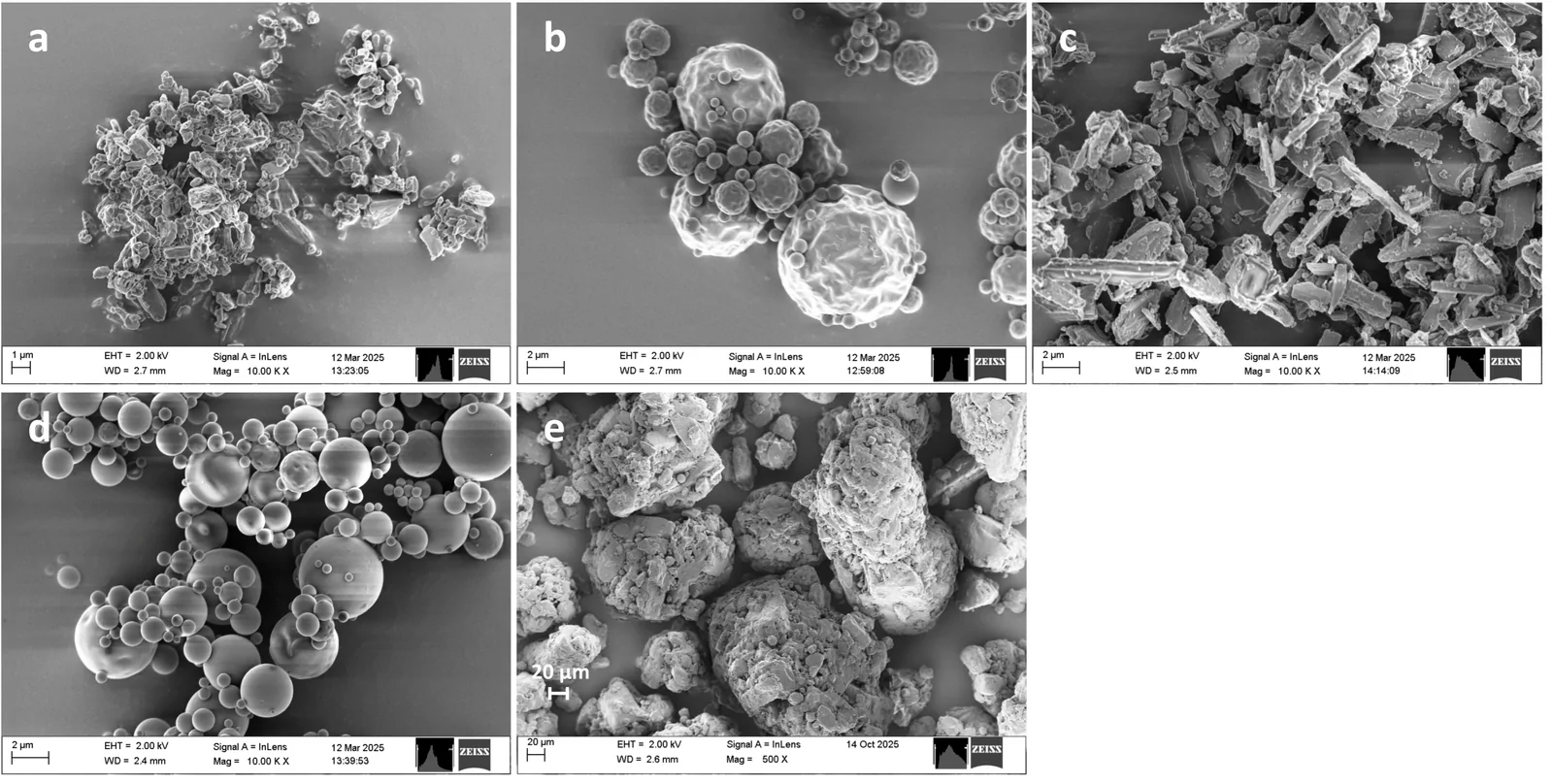
SEM images of all starting materials. (**a**) Micronized *TS*; (**b**) spray-dried *TS*; (**c**) micronized *SS*; (**d**) spray-dried *SS*; (**e**) lactopress.

Micronization and spray drying gave particles of distinctly different morphologies (Figure 2). The micronized *SS* particles were elongated with a needle-like morphology, while the micronized *TS* particles had more uniform dimensions but exhibited an angular character. For both drugs, the spray-dried particles had a spherical geometrical morphology. The *SS* particles had a smooth external surface while the *TS* particles had an uneven buckled surface structure. The Lactopress particles (the carrier) were slightly irregular and had an uneven surface texture.

For the respective drugs (*SS* and *TS*), the spray-dried particles had somewhat lower apparent particle density (*ρ*_p_) than the micronized drugs (Table 2). The median particle diameter *(D*_50_) of all drug particles was approximately 2 µm, with limited variation between particle types (1.89–2.39 µm), although the two largest diameters were observed for *SS* particles. Both types of *TS* particles had a wider particle size distribution (higher span) than the *SS* particles, due to a lower *D*_10_ for *TS* particles, i.e., about 0.4 µm compared with 1 µm. The micronized powders showed lower *ρ*_b_ than the spray-dried powders; however, for the respective particle type (micronized or spray-dried), similar powder packing densities were obtained. The *HR* of the two spray-dried materials was the same (1.67 for both), whereas for the micronized powders, *SS* had a lower *HR* than the spray-dried powders, while the micronized *TS* powder had a very high *HR*.

The spray-dried drug materials had similar values of *τ*_0_ (Table 3) and *ff*_c_. The micronized *TS* powder had higher *τ*_0_ and lower *ff*_c_ than micronized *SS* powder. Regarding the *φ*, the spray-dried materials had lower values than their respective micronized materials; however, both types of *TS* materials gave the highest *φ* overall.

Lactopress had, as expected, a markedly higher *D*_50_ and *ρ*_p_ than the drugs. Consequently, Lactopress powder had a low *HR*, a low *τ*_0_ and a high *ff*_c_ compared with the drug powders. The *φ* was lower for Lactopress powder than for the drug powders.

### 3.2. Appearance of Adhesive Mixtures

All adhesive mixtures of micronized and spray-dried drugs were examined using light microscopy. Based on this examination, it was concluded that at the lowest drug load, mixtures of both spray-dried and micronized drugs had a similar appearance, with drug particles located mainly within carrier cavities. As the drug load increased, an enveloped adhesive layer was formed for both spray-dried and micronized drugs, and the drug particles were thus attached to both cavities and the enveloped surfaces. The spray-dried particles tended to give a more voluminous adhesive layer located at the enveloped surfaces than the micronized particles. At a drug concentration of 20.0%, self-agglomerates of drug particles were visible not only under the microscope but also to the naked eye.

**Figure 3:**
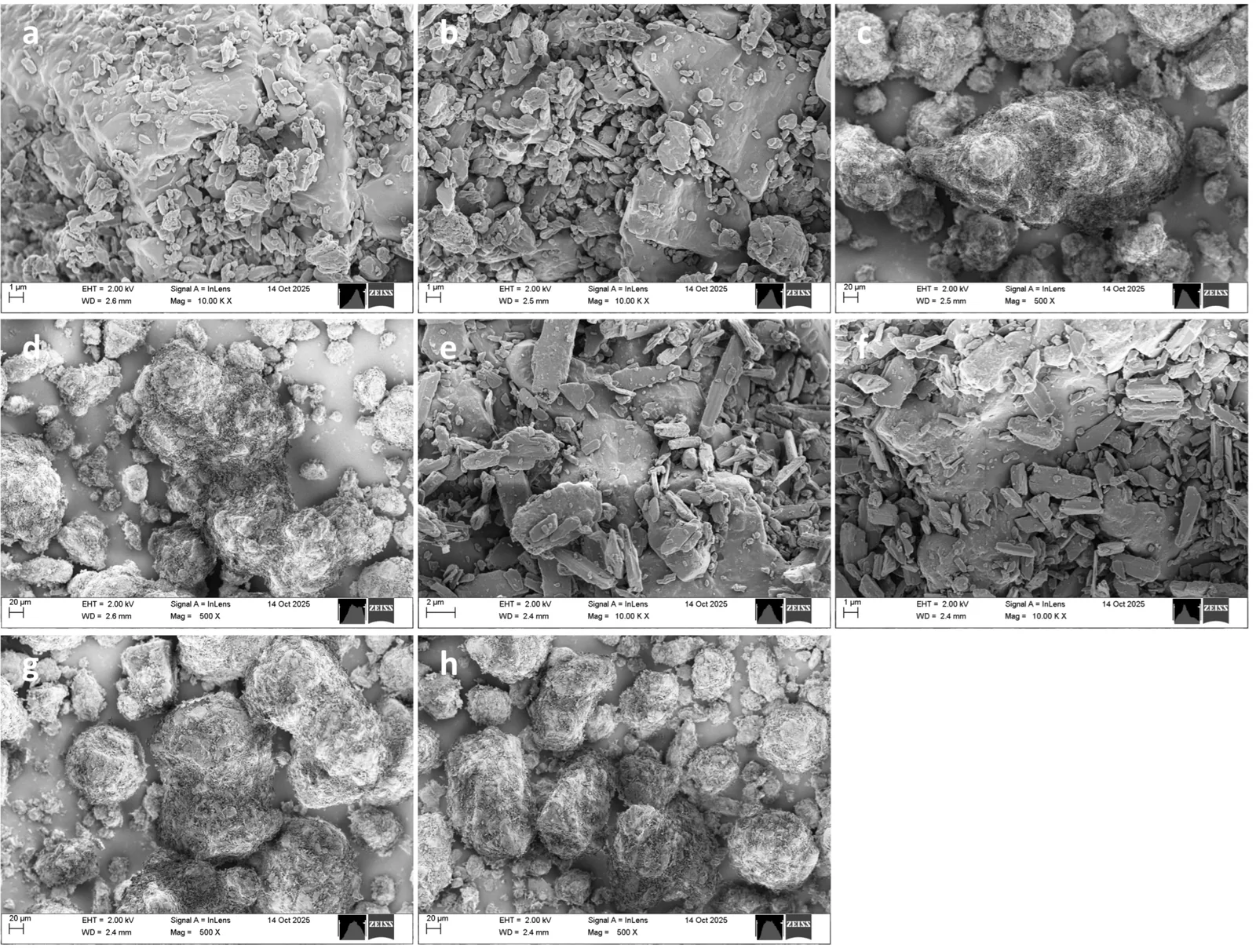
SEM images of adhesive mixtures of 3.85% (*w*/*w*) drug concentration. (**a**–**d**) Micronized *TS*; (**e**–**h**) micronized *SS*.

**Figure 4:**
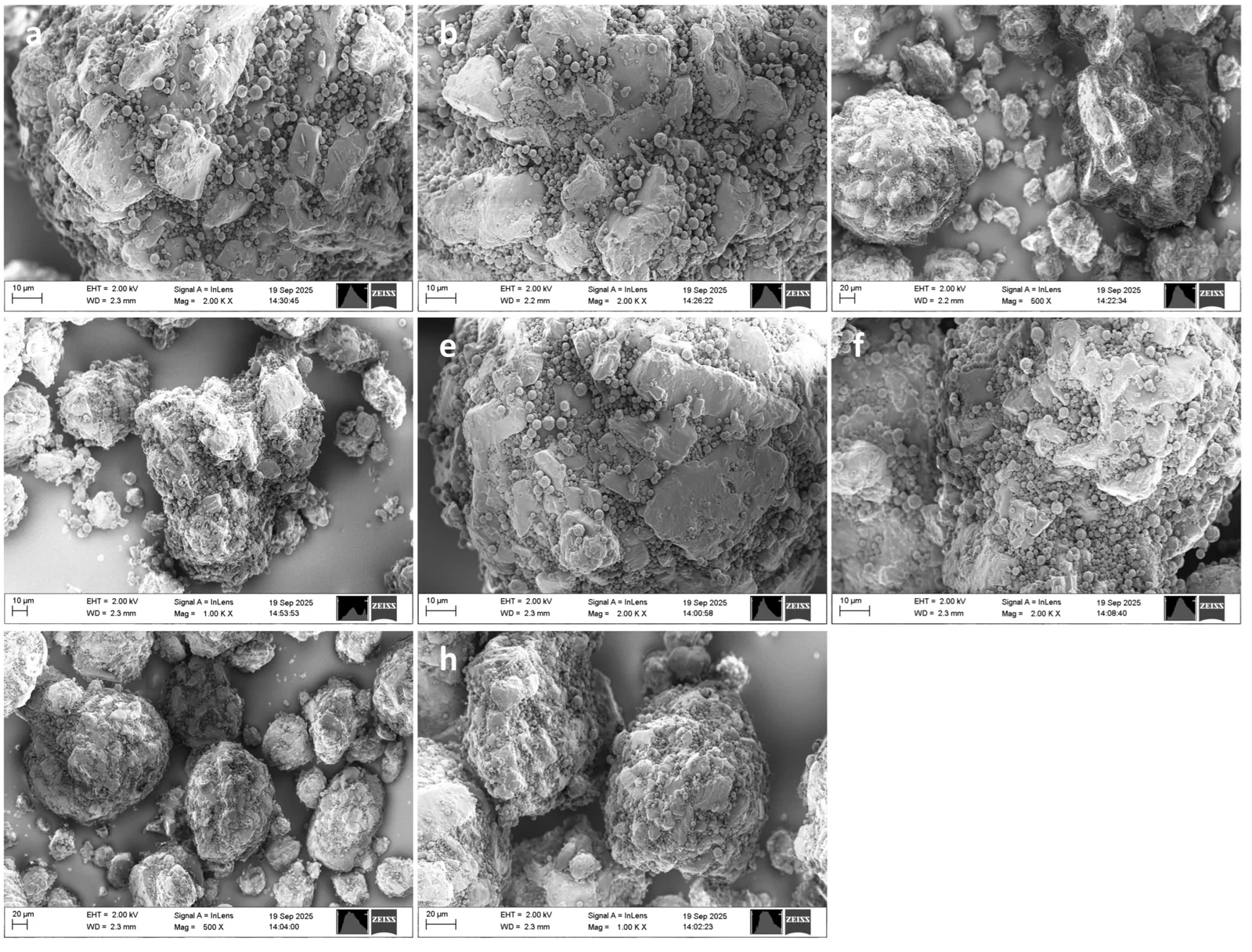
SEM images of adhesive mixtures of 3.85% (*w*/*w*) drug concentration. (**a**–**d**) Micronized *SS*; (**e**–**h**) spray-dried *SS*.

**Figure 5:**
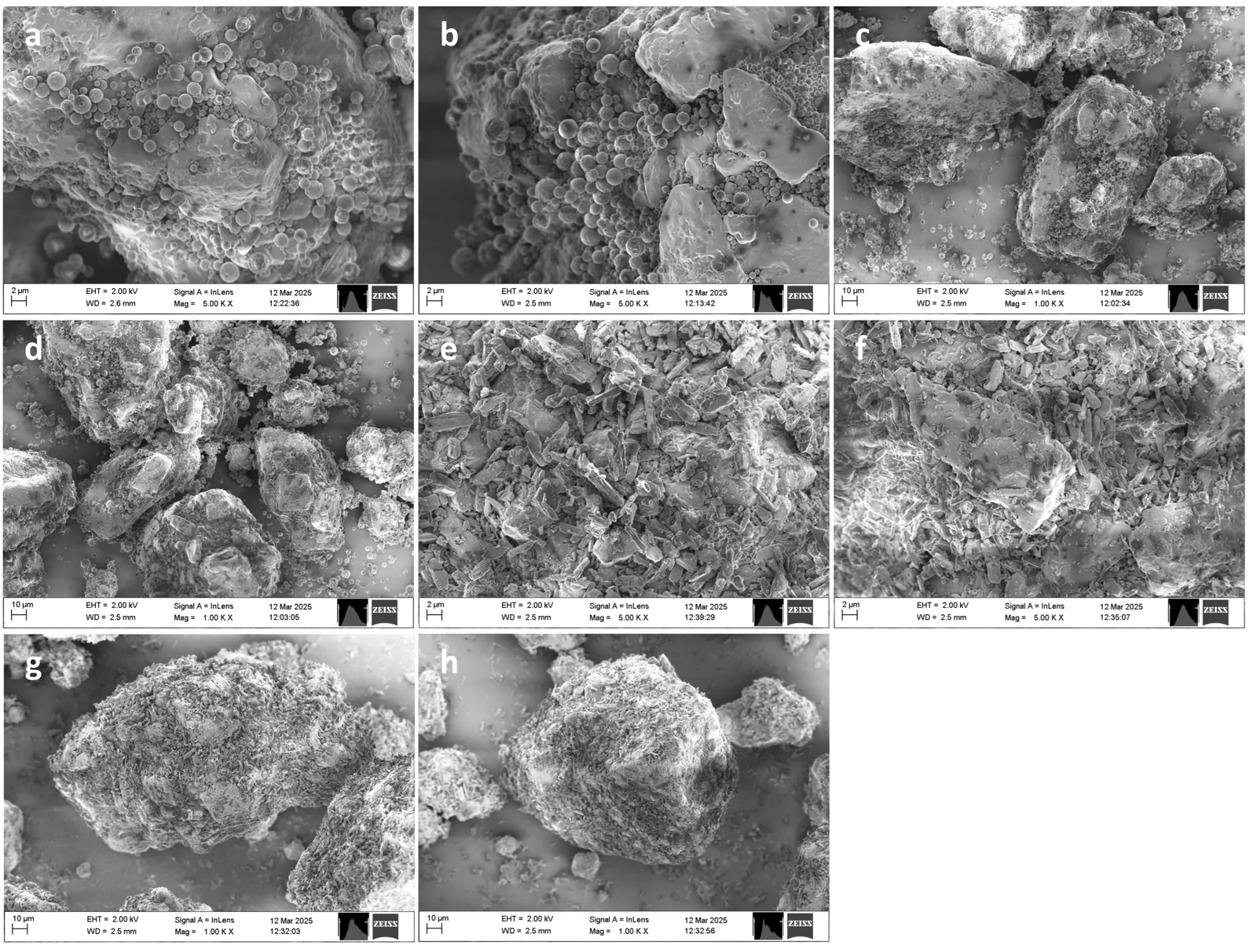
SEM images of adhesive mixtures of 13.0% (*w*/*w*) drug concentration. (**a**–**d**) Spray-dried *TS*; (**e**–**h**) micronized *SS*.

To illustrate this evolution in blend architecture with drug load, representative SEM images of adhesive mixtures at two drug concentrations, i.e., 3.85% and 13.0% *w*/*w,* are presented in Figure 3, Figure 4 and Figure 5. These concentrations were regarded as close to the drug loads corresponding to blend state 1 and blend state 2b. At 3.85% drug load, the drug particles were thus localized mainly in the carrier cavities, independent of the preparation process of the drug particles (micronized or spray-dried). The difference in particle shape between the micronized and spray-dried particles is clearly visible.

At a drug concentration of 13.0% *w*/*w* (Figure 5), drug particles were in addition attached to the enveloped carrier surface, with a tendency to form clusters. The spray-dried materials appeared to form a more voluminous adhesion layer, while the micronized materials appeared to form a more evenly distributed layer with a reduced tendency to form clusters or micro-agglomerates of attached particles.

### 3.3. Packing and Flow Properties of Adhesive Mixtures

**Figure 6:**
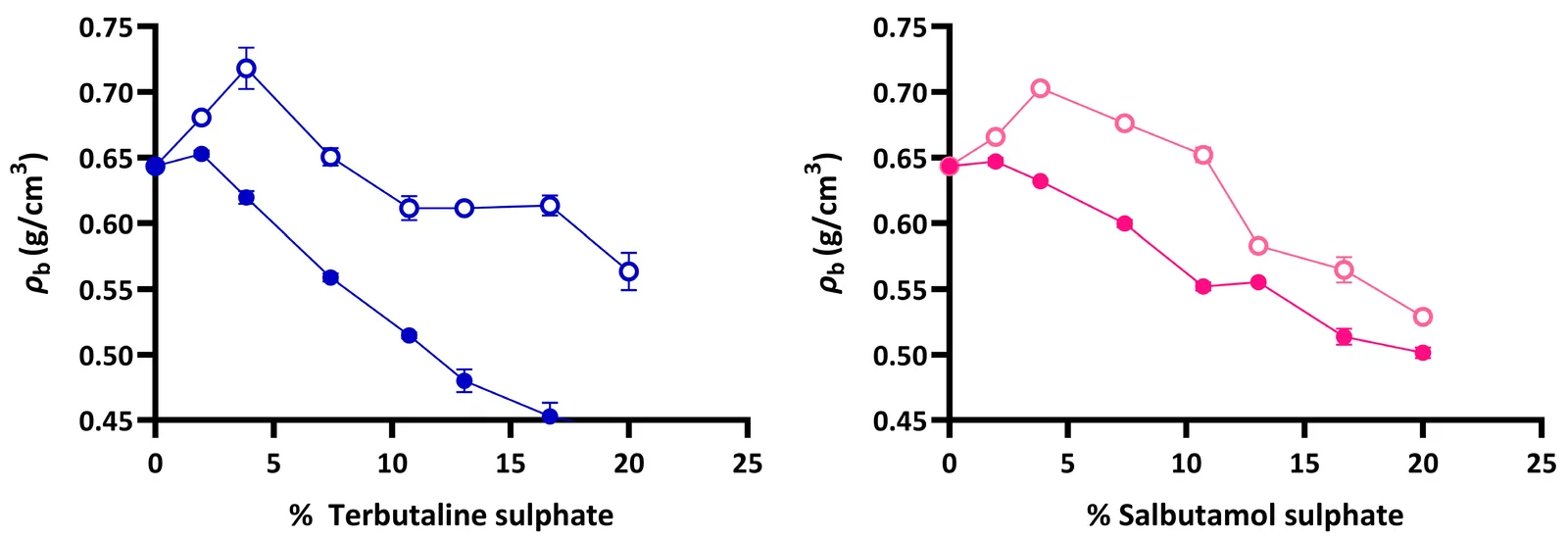
Effect of drug concentration (*w*/*w*) on the unconsolidated powder bulk density (*ρ*_b_). Closed symbols represent mixtures containing micronized drugs and open symbols represent mixtures containing spray-dried drugs. Mean values and standard deviations, n = 3.

The admixing of spray-dried drugs to the carrier gave at drug loads up to 4% a marked increase in *ρ*_b_ (Figure 6). Thereafter, a gradual but non-linear decrease in *ρ*_b_ was observed with increased drug load. For the micronized drugs, only a small increase in blend *ρ*_b_ was observed at 2% drug load, followed by an almost continuous decrease in blend *ρ*_b_ with further increases in drug concentration. A similar rate of reduction in *ρ*_b_ with drug load after the *ρ*_b_ peak was obtained for micronized and spray-dried drug powders.

**Figure 7:**
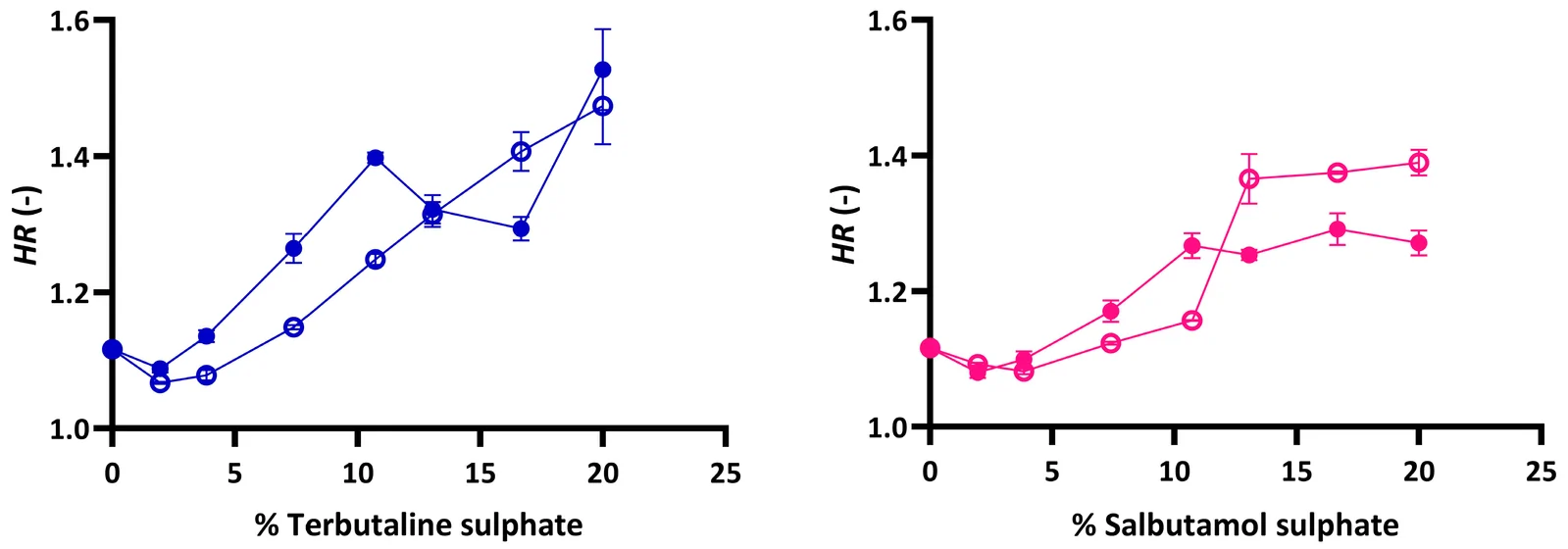
Effect of drug concentration (*w*/*w*) on the Hausner ratio (*HR*). Closed symbols represent mixtures containing micronized drugs and open symbols represent mixtures containing spray-dried drugs. Mean values and standard deviations, n = 3.

For all mixtures, the *HR* initially decreased slightly with increasing drug load, followed by a gradual increase (Figure 7). However, for the mixtures containing micronized *TS,* the *HR*-drug load relationship showed a second minimum at a drug load of 17%. The *TS* mixtures were generally more compressible than the *SS* mixtures as shown by their generally higher *HR* values. Regarding the differences between micronized and spray-dried materials, mixtures containing micronized drug showed higher values of *HR* than those containing spray-dried drug up to a drug concentration of 13%, at which point there was a shift, and spray-dried drug mixtures started to produce higher values.

**Figure 8:**
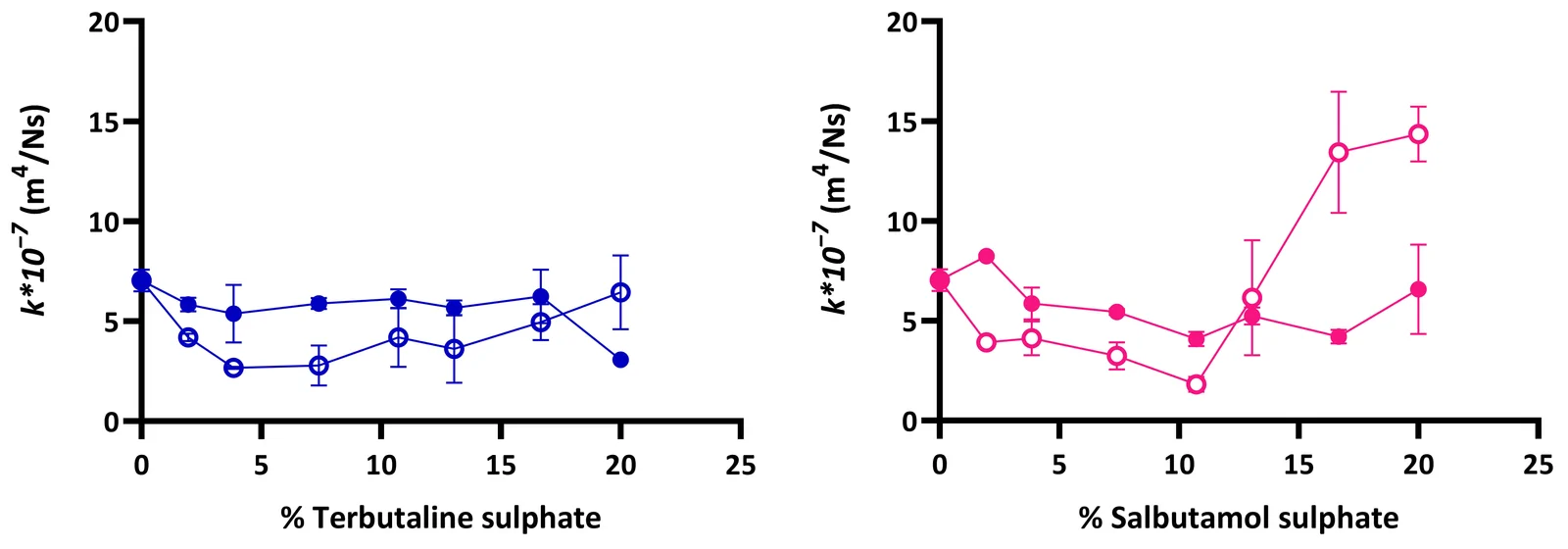
Effect of drug concentration (*w*/*w*) on the unconsolidated powder permeability (*k*). Closed symbols represent mixtures containing micronized drugs, and open symbols represent mixtures containing spray-dried drugs. Mean values and standard deviations, n = 3.

Mixtures containing micronized *SS* and *TS* powders exhibited fluctuating *k*-drug load relationships, with relatively small variations in *k* with drug load (Figure 8). In contrast, the gradual admixing of spray-dried drugs initially reduced *k*, followed by an increase at intermediate drug concentrations. For mixtures prepared with spray-dried *SS*, a distinct increase in *k* was observed at a drug load of 10%, and the *k* thereafter increased significantly up to 20% drug load.

**Figure 9:**
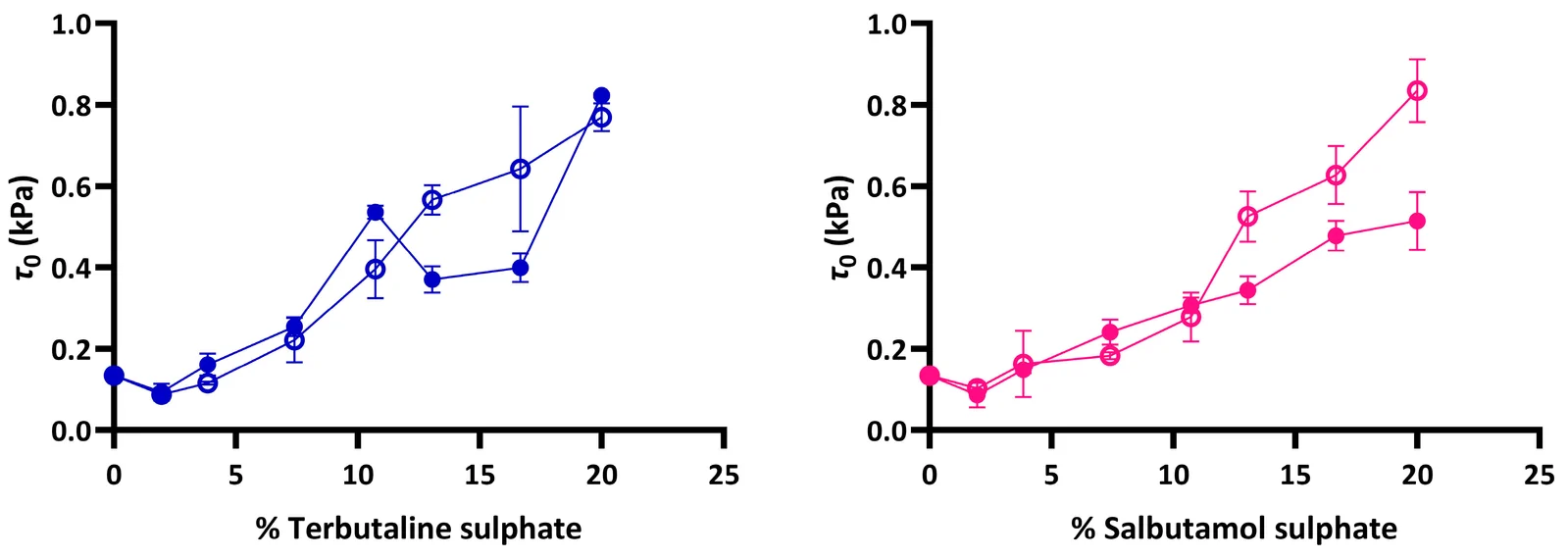
Effect of drug concentration (*w*/*w*) on the cohesion strength (*τ*_0_). Closed symbols represent mixtures containing micronized drugs and open symbols represent mixtures containing spray-dried drugs. Mean values and standard deviations, n = 3.

**Figure 10:**
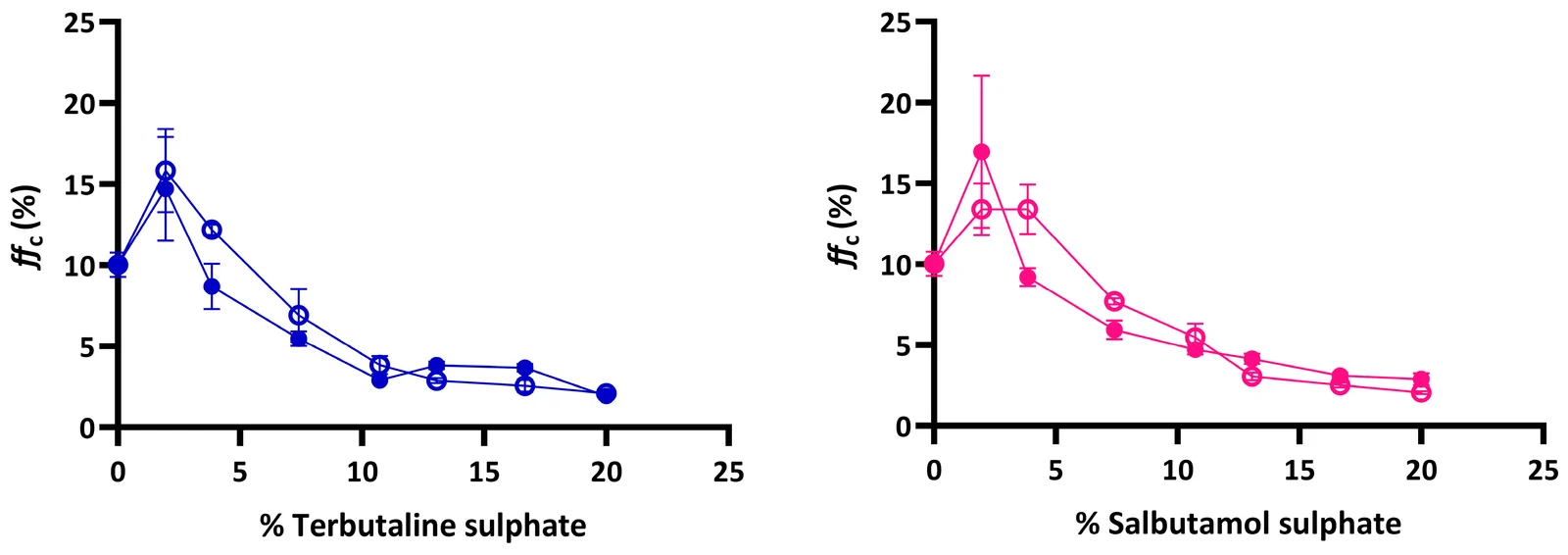
Effect of drug concentration (*w*/*w*) on the flow function coefficient (*ff*_c_). Closed symbols represent mixtures containing micronized drugs and open symbols represent mixtures containing spray-dried drugs. Mean values and standard deviations, n = 3.

**Figure 11:**
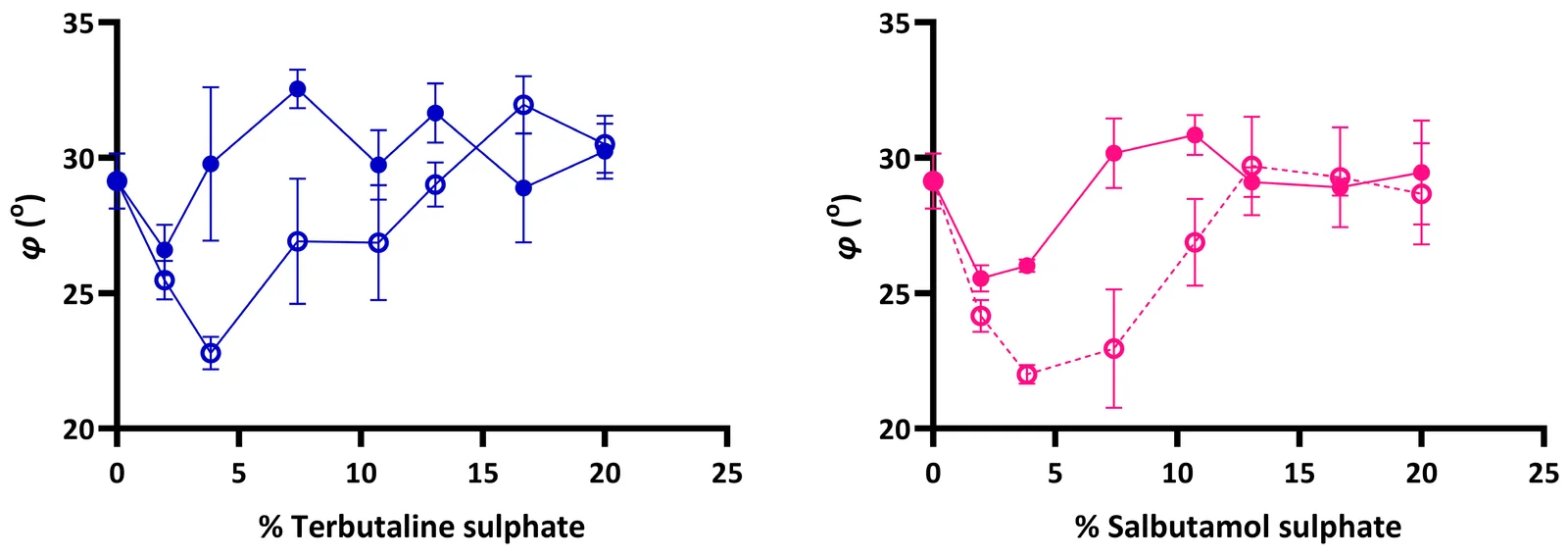
Effect of drug concentration (*w*/*w*) on the angle of internal friction (*φ*). Closed symbols represent mixtures containing micronized drugs and open symbols represent mixtures containing spray-dried drugs. Mean values and standard deviations, n = 3.

In Figure 9, Figure 10 and Figure 11, the influence of drug load on the evolution of the shear test parameters is shown. The *τ*_0_ showed a slight initial decrease for all adhesive mixtures, followed by a gradual increase (Figure 9). For the respective drug, mixtures containing micronized and spray- dried particles exhibited similar *τ*_0_ values up to a drug load of approximately 10%, after which the mixtures containing spray-dried materials displayed higher values (same phenomenon as seen for the *HR*). The most complex *τ*_0_—drug load relationship was observed for mixtures of micronized *TS* powder, similar to the trend seen for the *HR*.

The *ff*_c_ increased from approximately 10% (only carrier) to about 15% at the lowest drug load (2%) for mixtures containing all drug powders (Figure 10). Thereafter, the *ff*_c_ decreased non-linearly with increasing drug load, following a similar trend for all types of drug particles, with a final *ff*_c_ of about 2%. Although the profiles generally overlapped, mixtures containing spray-dried drugs tended to exhibit slightly higher *ff*_c_ values than those containing micronized drugs at low to intermediate drug loads (up to about 10%). At drug concentrations above approximately 10%, *ff*_c_ values were nearly constant for all mixtures.

The relationship between *φ* and drug load had for all drug types (Figure 11) a similar evolution. Initially, U-shaped relationships were obtained, which finally leveled out, i.e., at the highest drug concentrations, *φ* was nearly constant in a similar way as for *ff*_c_. Mixtures containing spray-dried powders exhibited a stronger initial decrease in *φ* for both drugs, but the final *φ* values were similar across all mixtures.

### 3.4. Correlations Between Packing and Flow Properties

**Figure 12:**
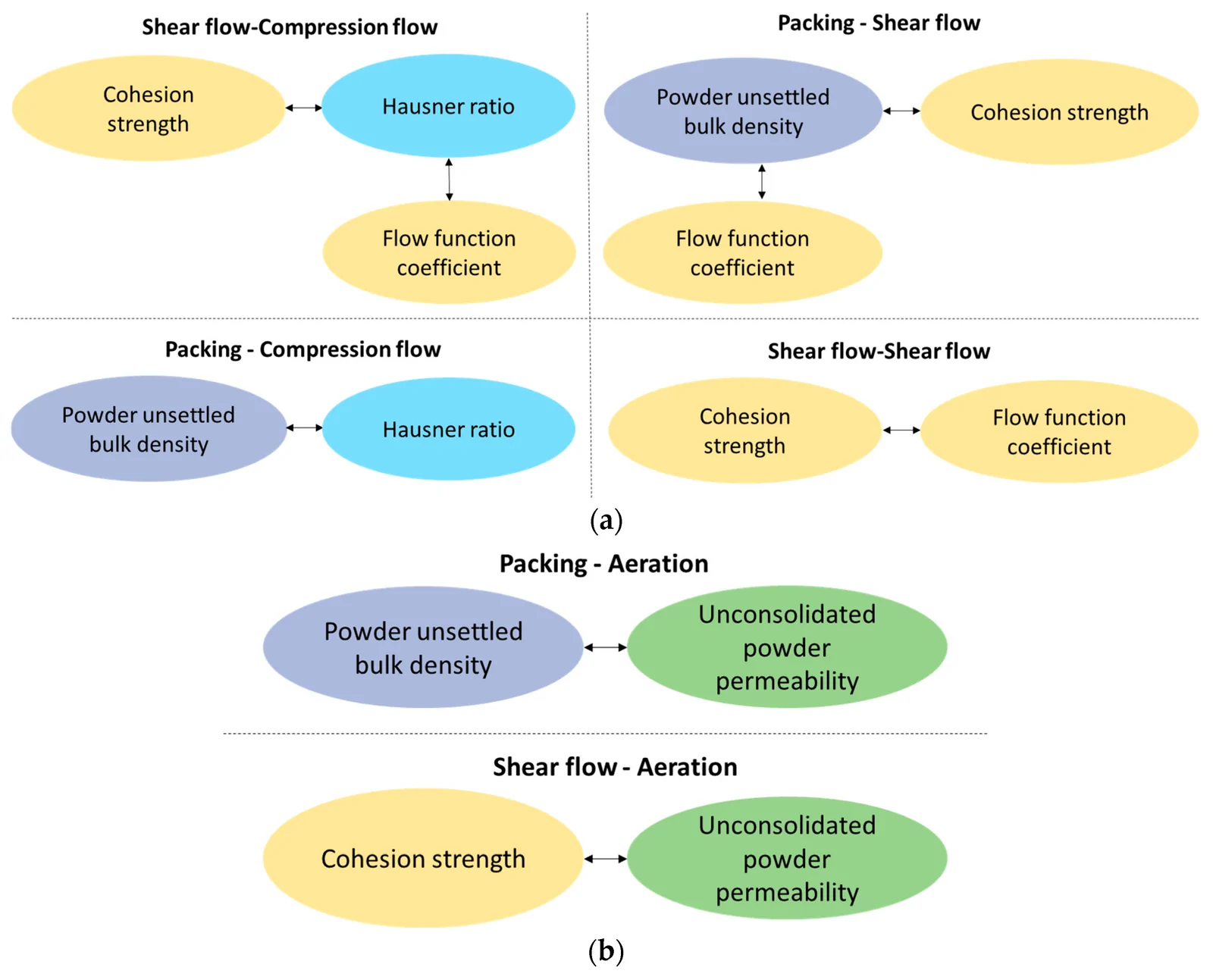
(**a**). Significant correlations from the Pearson analysis of packing and flow properties for all mixtures of micronized and spray-dried *TS* and *SS* (*p* < 0.01). (**b**). Additional significant correlations from the Pearson analysis of packing and flow properties for mixtures of spray-dried *SS* (*p* < 0.01).

The evolution pattern of the different measures of packing and flow properties was, as reported above, dependent on the measurement method used. In order to assess if any measures nevertheless correlated to each other, a Pearson analysis was done, and the outcome is presented in four groups in Figure 12a,b, i.e., in accordance with an earlier paper [19]. A series of significant correlations were in fact obtained for measures of both packing density and flow properties. The highest number of significant correlations was obtained for mixtures containing spray-dried *SS* powder (Figure 12b). One can note that no significant correlation was observed for the *φ* with any other mechanical parameter.

### 3.5. In Vitro Aerosolization of Adhesive Mixtures

The impactor (NGI) experiments were performed on a restricted number of mixtures, i.e., blends of four drug loads covering a broad range of drug concentrations and thus blend states. The *FPF* is a simple in vitro measure of the aerosolization of an inhalation powder and thus has limitations [29]. It has nevertheless emerged as an important in vitro indication of the potential lung dose and is here calculated from the emitted dose (*ED*) and the *FPD*. In addition to the dose measures, we here also report the *MMAD* as an indication of the dispersion of the mixtures.

**Figure 13:**
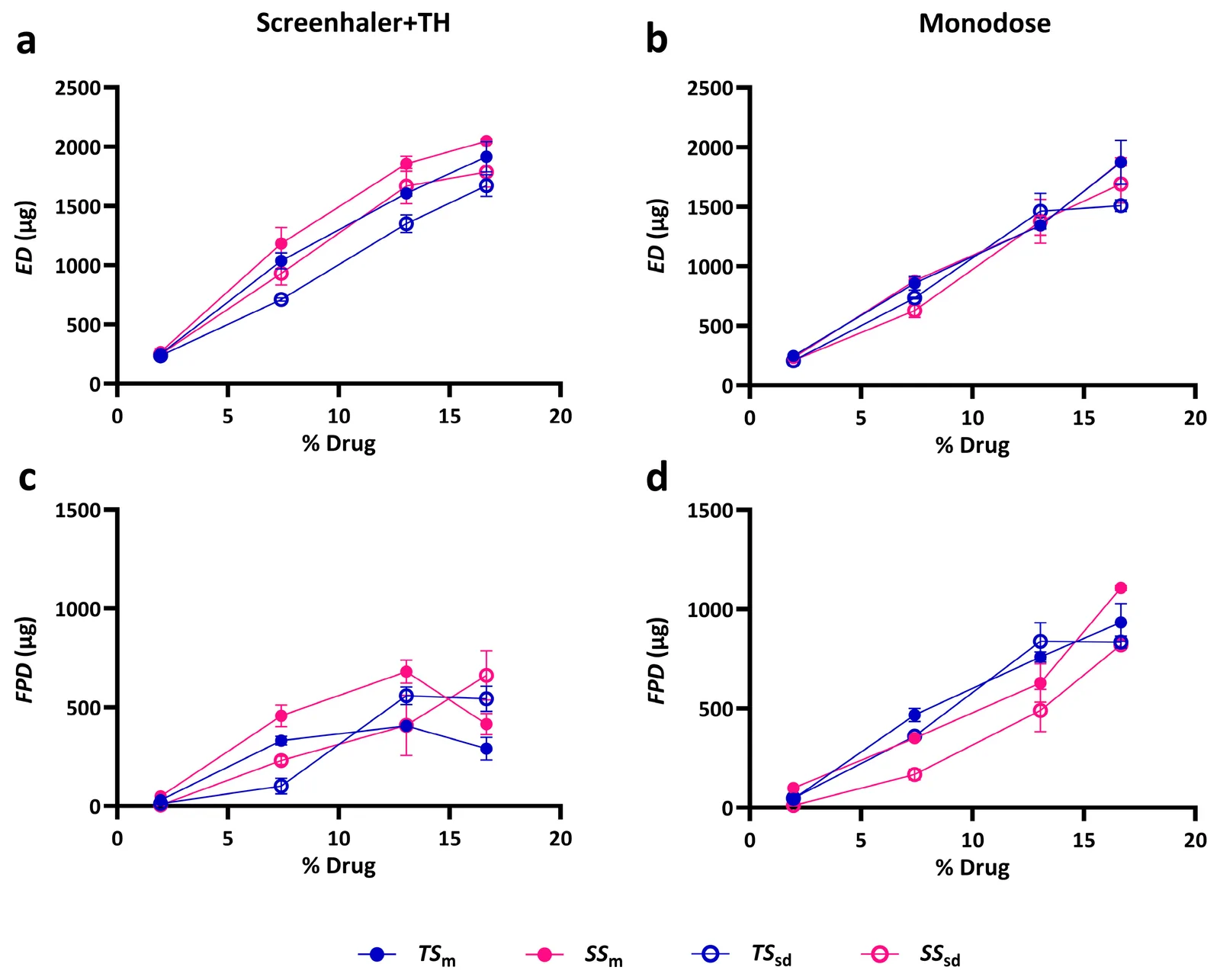
Effect of drug concentration (*w*/*w*) on the emitted dose (*ED*) and fine particle dose (*FPD*) for the four mixtures used in the impactor experiments. (**a**) *ED* using the Screenhaler device equipped with a Turbuhaler mouthpiece; (**b**) *ED* using the Monodose device; (**c**) *FPD* using the Screenhaler device equipped with a Turbuhaler mouthpiece; (**d**) *FPD* using the Monodose device. Subscripts m and sd represent micronized and spray-dried drug, respectively. Mean values and standard deviations, n = 3.

For both inhalers, the *ED* increased almost linearly with drug load, ranging from approximately 200 to 1900 µg (Figure 13). For the Screenhaler, the micronized materials yielded slightly higher *ED*s than those containing spray-dried drug, except at the lowest drug load where all mixtures produced comparable *ED*. In contrast, for the Monodose device, the *ED*–drug load relationships for both types of materials overlapped across the whole range of drug concentration used.

Both the solid form of the drug material and the type of device influenced the *FPD*-drug load relationship (Figure 13). The Screenhaler device gave bent or arch-shaped *FPD*—drug load relationships while the Monodose device gave nearly linear relationships. For both devices, all drug types yielded a low and nearly the same *FPD* at the lowest drug load. Thereafter, *FPD*-drug load relationships diverged except for micronized and spray-dried *TS* powder using the Monodose device. Generally, the spray-dried powders tended to give lower *FPD*s than the micronized powders.

**Figure 14:**
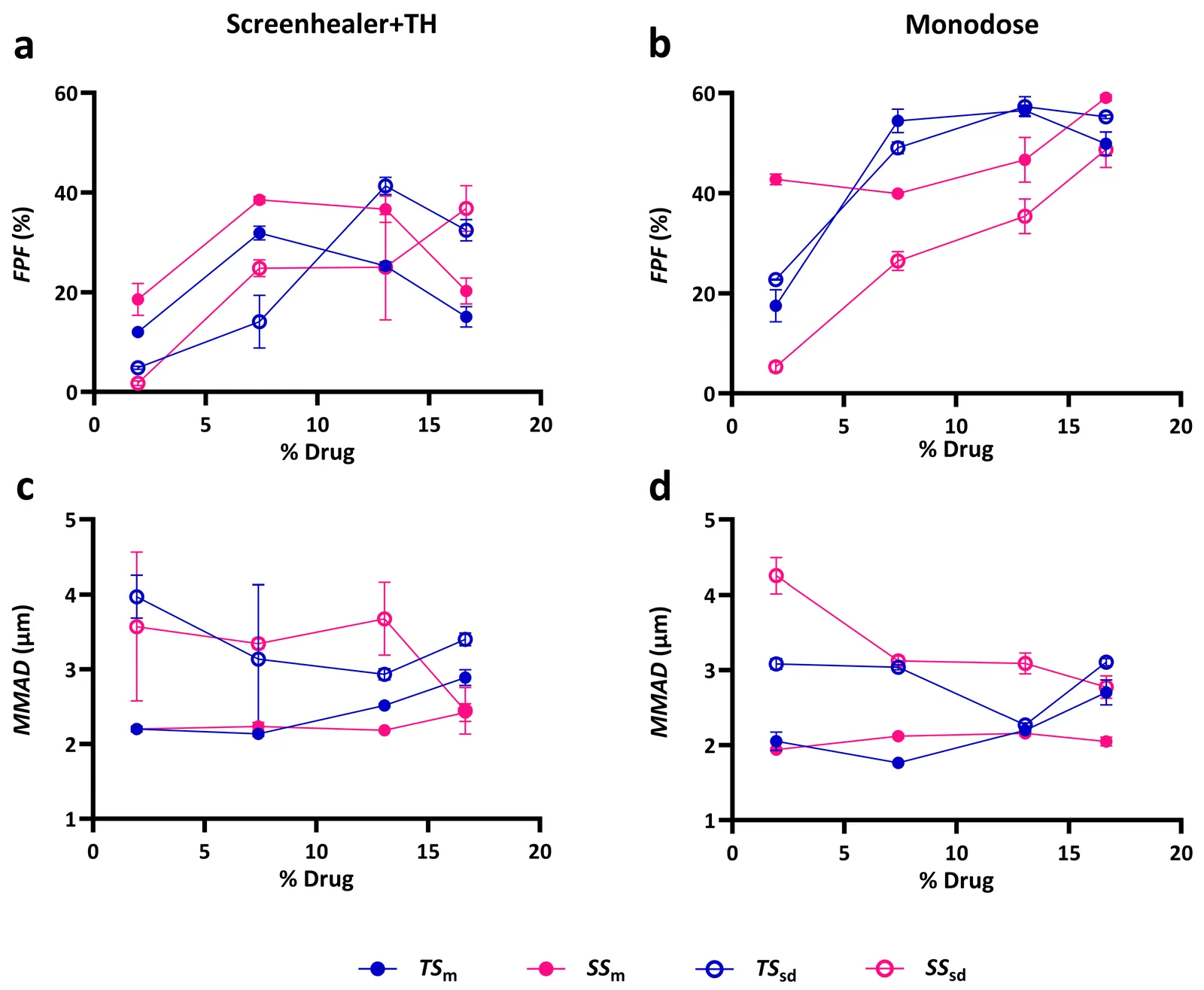
Effect of drug concentration (*w*/*w*) on the fine particle fraction (*FPF*) and the mass median aerodynamic diameter (*MMAD*) for the four mixtures used in the impactor experiments. (**a**) *FPF* using the Screenhaler device equipped with a Turbuhaler mouthpiece; (**b**) *FPF* using the Monodose device; (**c**) *MMAD* using the Screenhaler device equipped with a Turbuhaler mouthpiece; (**d**) *MMAD* using the Monodose device. Subscripts m and sd represent micronized and spray-dried drug, respectively. Mean values and standard deviations, n = 3.

The progression of the *FPF*-drug load relationships was more or less complicated depending on the combination of device and drug type (Figure 14). The Screenhaler device gave arch-shaped *FPF*-drug load relationships for mixtures containing micronized drugs, while the spray-dried drugs produced fluctuating relationships without an arch-shape. The Monodose device gave either overlapping bent relationships toward a leveling-off (*TS* mixtures) or nearly linear or slightly curved, clearly separated relationships (*SS* mixtures). Regarding the effect of the device, the Monodose generally produced higher *FPF*s than the Screenhaler. Regarding the solid form of the drug, the micronized drugs tended to yield higher *FPF*s than the spray-dried drugs except at the highest drug load. Finally, the chemical drug compound did not give a consistent effect on the *FPF*.

The *MMAD* ranged between 2 and 4 µm for both devices (Figure 14), i.e., the device design had only a limited effect on *MMAD*, in contrast to its influence on the *FPF*. Moreover, drug load had only a minor effect on *MMAD*; however, there were differences depending on the composition of the mixtures. Specifically, mixtures containing spray-dried drugs tended to yield higher *MMAD*s than those containing micronized drugs.

### 3.6. Correlations Between Mechanical and Aerosolization Properties

**Figure 15:**
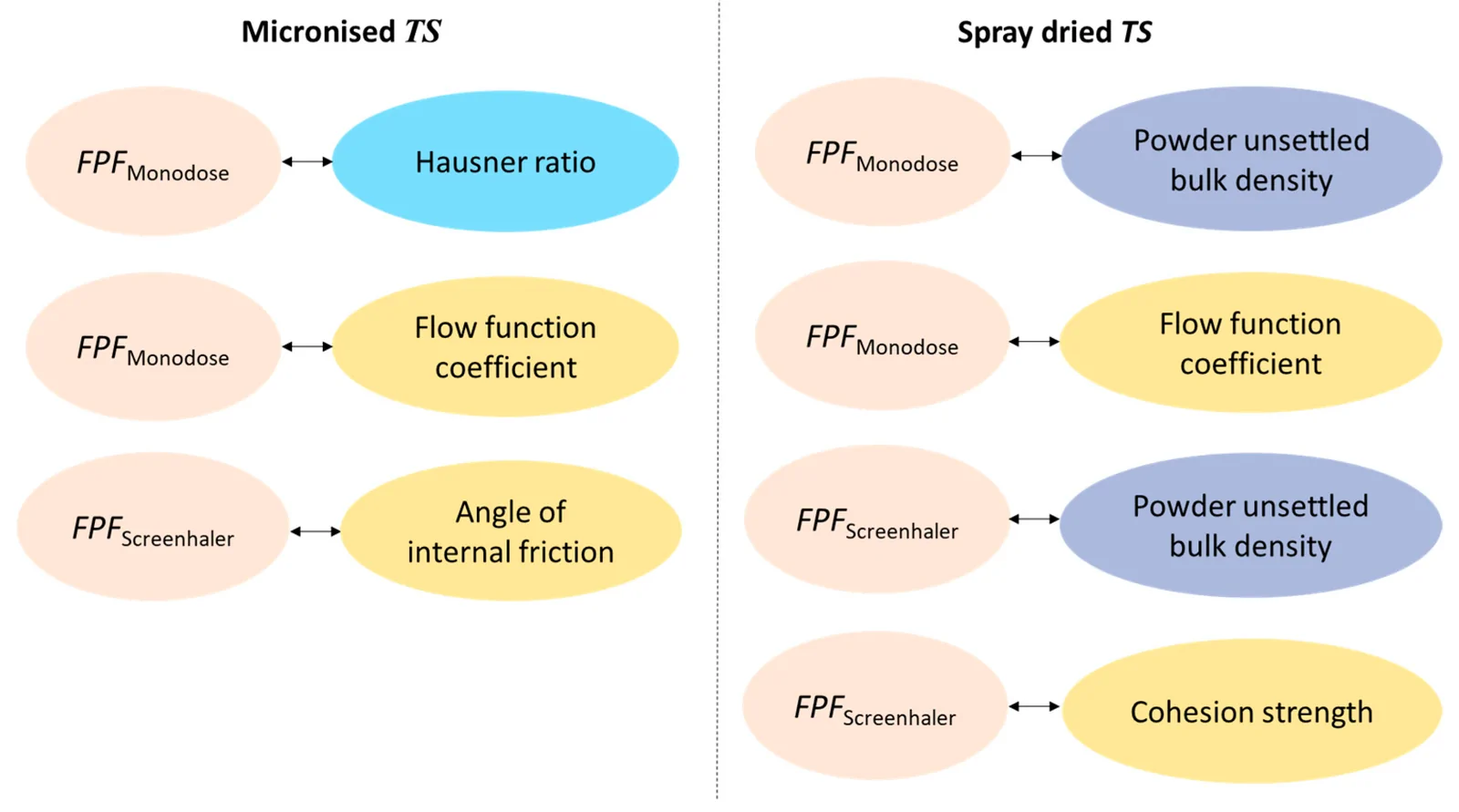
Correlations from the Pearson analysis of packing and flow properties on one hand and *FPF* on the other (*p* < 0.1) of mixtures of micronized and spray-dried *TS*.

**Figure 16:**
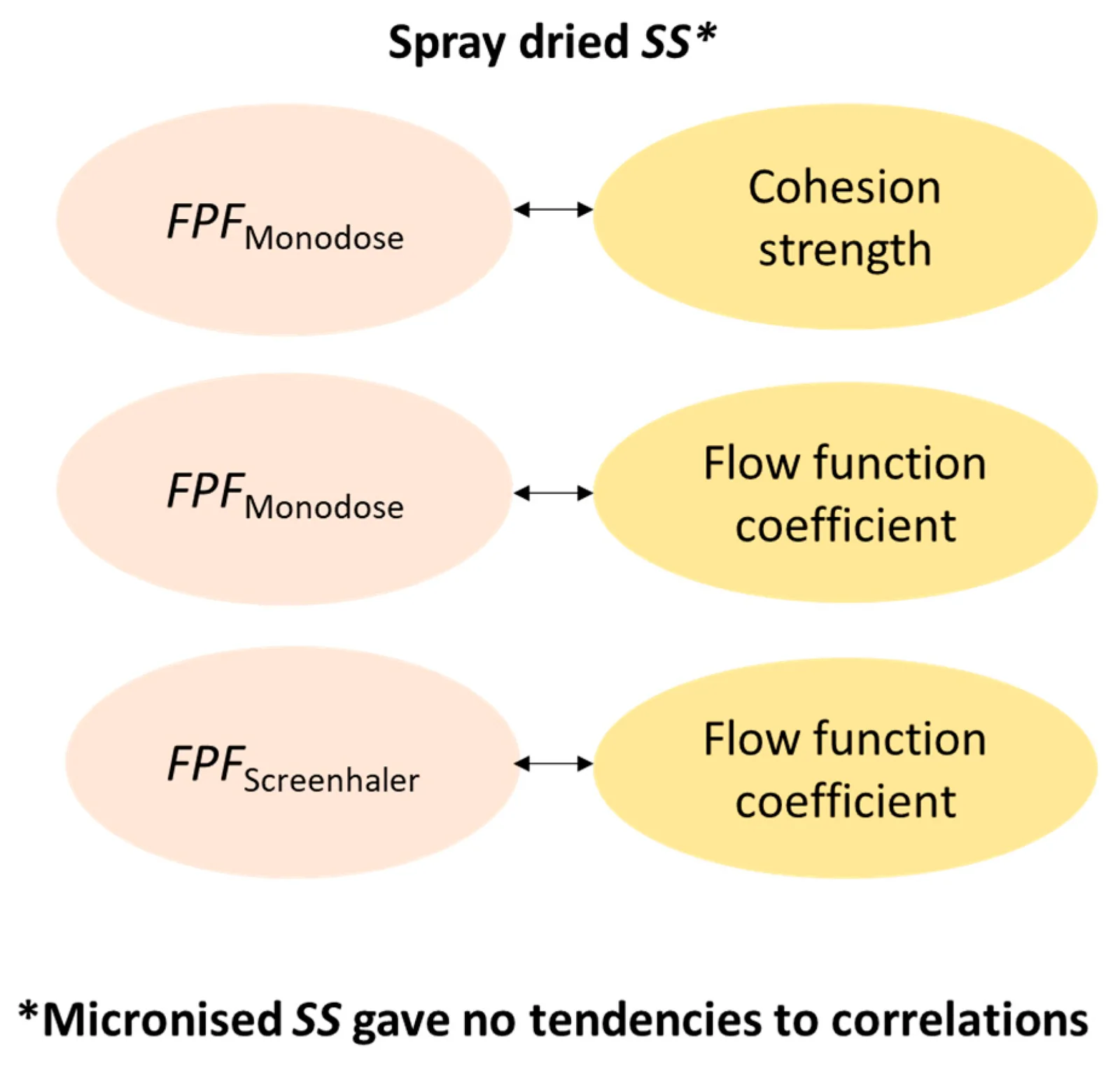
Correlations from the Pearson analysis of packing and flow properties on one hand and *FPF* on the other (*p* < 0.1) of mixtures of spray-dried *SS*. *N.B.* Micronized *SS* gave no tendencies to correlations.

Unlike the packing and flow properties, for which a series of significant Pearson correlations were common across all mixtures, correlations between packing and flow properties, on the one hand, and aerosolization propensity, on the other (Figure 15 and Figure 16) were few. The lack of correlations may partly be explained by the limited number of drug concentrations used in the aerosolization experiments. There were no correlations between *MMAD* and the mechanical properties of the mixtures, but the *FPF* tended to correlate predominantly with measures of the shearing properties of the powders (see also Table A1 and Table A2). Moreover, the correlations observed were also dependent on the device, highlighting the complexity of the aerosolization process.

## 4. Discussion

### 4.1. Comments on Experimental Design

In this paper, binary adhesive mixtures of a wide range of concentrations of micronized and spray-dried drug particles have been studied. The preparation of the two types of drug particles aimed to achieve three goals: First, particles of different crystallinity; second, particles of different shapes; and third, inhalable particles of similar particle diameter distributions. It is concluded that the goals of the drug particle preparations were fulfilled. Lactopress was chosen as carrier since it was earlier shown that adhesive mixtures prepared with this carrier [10] display a distinct transition from blend state 1 to blend state 2.

Since amorphous particles are inherently unstable, the *RH* at room temperature at which the powders are stored and handled must not exceed a critical level to avoid recrystallization. In this study, the micronized drug powders and mixtures were generally stored and handled at a *RH* of 30–35%, while the spray-dried drug powders were stored at 1% *RH*.

The drug concentration of the mixtures used in this study varied between 2–20 wt.%. Grasmeijer et al. [12] reported that the drug concentration in adhesive mixtures used in marketed products lies in the range of 0.1 to 4%, and the maximum drug concentration that can typically be used in adhesive mixtures has been stated to be about 10% [13,30]. Above the 10% level, self-agglomerates of the drug may be formed [31].

The weight of an adhesive mixture filled into commercial inhalers is typically in the range 10–25 mg [31], and we here used 15 mg of mixture for each actuation. For pulmonary drug delivery, a nominal drug dose [30] or a total lung dose [31] below 1 mg is typically considered a low drug dose, and the nominal dose range used in this study (0.3–3 mg) corresponds thus to low and intermediate pulmonary drug doses.

In an earlier study [19], the methods used for assessing the packing and flow properties of powders were divided into six classes, and tests from four of these classes were used. In this study, the same four classes of tests were used to provide a broad representation of the packing and flow properties of the adhesive mixtures.

To investigate whether the inhaler type affected the dispersion performance of the mixtures, two inhalers were used: a Screenhaler device [26] and a capsule-based device (Monodose). In the Monodose inhaler, a size 3 capsule was used, which is the standard size in capsule-based inhalers [32]. The Screenhaler is simply a bent tube into which the mixture to be aerosolized is poured and subsequently pushed away from its residence site during actuation. In this study, the device was equipped with a Turbuhaler mouthpiece to facilitate the dispersion of the mixtures [33]. In the Monodose inhaler, the capsule spins during actuation, and the powder is released from the rotating capsule through pierced holes. Eventually, the powder leaves the chamber, passes through a grid, and is transported out of the inhaler via the mouthpiece. The forces to which the adhesive units are subjected during actuation are probably markedly higher in the Monodose device than in the Screenhaler. For both devices, the mixture was manually poured directly into the Screenhaler device or into the capsule before insertion into the inhaler. This ensured that an unconsolidated mixture bed was exposed to the flowing air in both devices.

The aerosolization study was conducted at a single pressure drop [34,35]. The Monodose device was operated at a volumetric airflow rate of 65 L/min, at which no remaining powder agglomerates in the capsules post-actuation were visually observed in our experiments. The Screenhaler device was operated at an airflow rate of 70 L/min, which has earlier been shown [11] to produce a *FPF* close to the maximum achievable.

### 4.2. Effect of Drug Load on Appearance and Mechanical Properties of Adhesive Mixtures

#### 4.2.1. Appearance

At low drug concentrations, drug particles were located and concentrated in surface irregularities of the carrier; at intermediate concentrations, they also adsorbed onto the carrier enveloped surface, forming a progressively thicker layer. At high concentrations, free self-agglomerates of drug particles were additionally formed. Thus, the drug concentration range used represented blend states 1, 2 and 3. Therefore, the blend state model appears to describe the development of the physical state of adhesive mixtures for both micronized and spray-dried materials. The formation of three stages of blend structure with increased fine concentration has also been reported earlier [5] for ternary adhesive mixtures, using fine lactose as a dispersion-enhancing excipient.

The spray-dried materials formed adhesive layers that appeared more voluminous with a more irregular outer surface. Spray-dried particles tend to form small agglomerates during spray drying, micro-agglomerates that may resist break-up during mixing with the carrier and thus become adsorbed onto the enveloped surface of the carrier as micro-agglomerates. Thus, the method of drug particle preparation affected the evolution of the microstructure of the adhesive layer.

#### 4.2.2. Packing

All four drug powders exhibited similar patterns for the relationship between *ρ*_b_ and drug concentration. The initial increase in *ρ*_b_ has previously [10] been attributed to the enrichment of drug particles in surface irregularities of the carrier, which increases the mass of a carrier particle with only a limited effect on its volume and packing density. Moreover, small amounts of adhered drug particles may reduce friction between the carrier particles (see discussion below) and promote a more closely packed structure. The subsequent formation of an adhesive layer on the enveloped surface of the carrier particles increased the separation distance between carriers and thereby reduced the overall packing density, i.e., the *ρ*_b_ decreased. This layer gradually thickens with increased drug load, resulting in a gradual decrease in *ρ*_b_.

The spray-dried materials generally produced higher mixture *ρ*_b_ across the whole range of drug loads due to a greater initial increase in *ρ*_b_. Thus, a larger mass of spray-dried particles could be accommodated within the surface irregularities of the corrugated carrier particles. Elsayed and co-workers [5] also reported that different types of fines in adhesive mixtures gave different *ρ*_b_-fines concentration relationships.

The spray-dried particles (Table 3) exhibited a lower *φ* than the corresponding micronized powder. The *φ* is an indication of the frictional or shear resistance of a powder, and a higher value of *φ* indicates a greater ability to withstand stress, i.e., increased mechanical stability of the powder. Therefore, the spray-dried particles had a lower ability to withstand stress and, when sheared and squeezed into carrier surface irregularities during mixing, were more prone to compress and form a denser packing structure. This higher compressibility may be due to the more regular, nearly spherical shape of the spray-dried particles (Figure 3). Moreover, it was earlier shown [36] that amorphous lactose particles are more plastically deformable than their crystalline counterpart. In analogy, we propose that a difference in plastic deformability existed between the amorphous and crystalline materials of the drugs used in this study, which also may contribute to a greater tendency of the softer amorphous particles to be compressed into surface cavities during mixing. These two particle properties are proposed as possible contributors, but their individual effects were not separated.

At low and intermediate drug concentrations, the adhesive mixtures exhibited similar or lower *k* than the carrier powders (Figure 9). It seems thus that when the drug particles were forced into carrier cavities, a more closely packed structure of the adhesive units was formed, which caused a decrease in *k*. Moreover, a limited fraction of drug particles may have been adsorbed onto the enveloped carrier surfaces even at low drug concentrations, increasing resistance to airflow and further reducing *k*. At high drug concentrations, mixtures of micronized *SS* showed a marked increase in *k*, whereas a similar effect was not seen for the other materials. For mixtures of spray-dried *SS*, a marked change in packing structure occurred at high drug load, which is proposed to be due to the formation of relatively large free self-agglomerates at a drug concentration of about 15%, which may open up the pore structure and increase the *k*.

#### 4.2.3. Flow

The statistical correlations obtained between the *HR*, *τ*_0_, and *ff*c indicate a similar progression of these flowability indications with increasing drug concentration (Figure 7, Figure 9 and Figure 10). At the lowest drug concentrations, flowability increased, consistent with previous observations that small amounts of fine particles can act as a glidant [19]. At intermediate and high drug concentrations, when an adhesive layer had formed on the enveloped carrier surfaces, flowability gradually decreased, and the adhesive layer thus acted as an anti-glidant [19]. Both the micronized and the spray-dried particles exhibited similar overall trends in the flowability-drug load relationships, but there was a tendency for the spray-dried particles to provide slightly better flowability at low and intermediate concentrations, despite the morphological differences of the enveloped adhesive layer described above. The adhesive layer on the carrier surface (i.e., blend state 2) started to form at higher drug load for the spray-dried particles; since this layer negatively affects flowability, the onset of reduced flowability was correspondingly delayed, i.e., shifted to higher drug concentrations.

At drug concentrations above 10%, the *HR* reached for most drugs a level of about 1.4, corresponding to low flowability. At this drug load, the outer surface of the adhesive layer became rough, which can increase the mechanical interlocking between the adhesive units with a consequent decrease in flowability.

The *φ* showed a different evolution with drug load than that of the other flowability parameters (Figure 12) and thus did not statistically correlate with any other measure of flowability [19]. This lack of correlation can at least be partly attributed to the high variability of the *φ* values. Nevertheless, the progression of *φ*-drug load relationships was similar for all types of drug particles. Mechanistically, this progression of the *φ*-drug load relationships can be linked to the structural changes of the adhesive layer with increased drug load. The addition of a low amount of drug decreased *φ* for all drug materials, more markedly for the spray-dried drugs. This glidant effect is a result of the filling of carrier surface cavities (active sites), which smooths the carrier surfaces and reduces the friction or shear resistance between the adhesive units. When the cavities are saturated, corresponding to the minimum of the *φ*-drug load relationships, drug particles start to adsorb relatively strongly onto the enveloped surface of the carrier (blend state 2a), creating a more uneven surface with an increased potential for interlocking between carriers, i.e., an anti-glidant effect. Shear resistance then increases, seemingly in proportion to the degree of area coverage of the enveloped carrier surface, until the carriers are covered with a thin layer of drug fines. Thereafter, a thicker and more voluminous adhesive layer gradually forms (blend state 2b), whose external structure may be independent of the thickness of the adhesive layer. Consequently, the frictional resistance levels out, becoming independent of the adhesive layer thickness. It can also be noted that the *ffc* tended to level out at the highest drug loads, approaching a flowability value largely independent of drug load.

Since the micronized powders of *TS* generally exhibited lower *ρ*_b_ and higher *HR* than the *SS* powders, it seems that micronized *TS* powders formed thicker adhesive layers in blend state 2, which gave a more compressible powder during loading. Regarding the spray-dried powders, the *SS* powder displayed markedly higher *k* at the two highest drug loads than the corresponding *TS* powders, which may be due to the formation of free self-agglomerates of the drug. These relatively limited effects of the drug substance are proposed to be attributed to differences in particle shape.

#### 4.2.4. Evolution of Blend Structure

In summary, for both substances studied, the evolution of the blend structure or architecture followed the consecutive steps of the blend state model. However, differences in the evolution of the blend structure between micronized and spray-dried particles were observed in three respects:

1. A larger mass of spray-dried particles was able to pack into depressions on the carrier surface, i.e., in blend state 1.
2. Spray-dried particles formed a more voluminous and irregular adhesive layer on the external surface of the carrier, i.e., in blend state 2.
3. Spray-dried particles formed free self-agglomerates at a lower concentration, i.e., they entered blend state 3 at a lower drug load.

**Figure 17:**
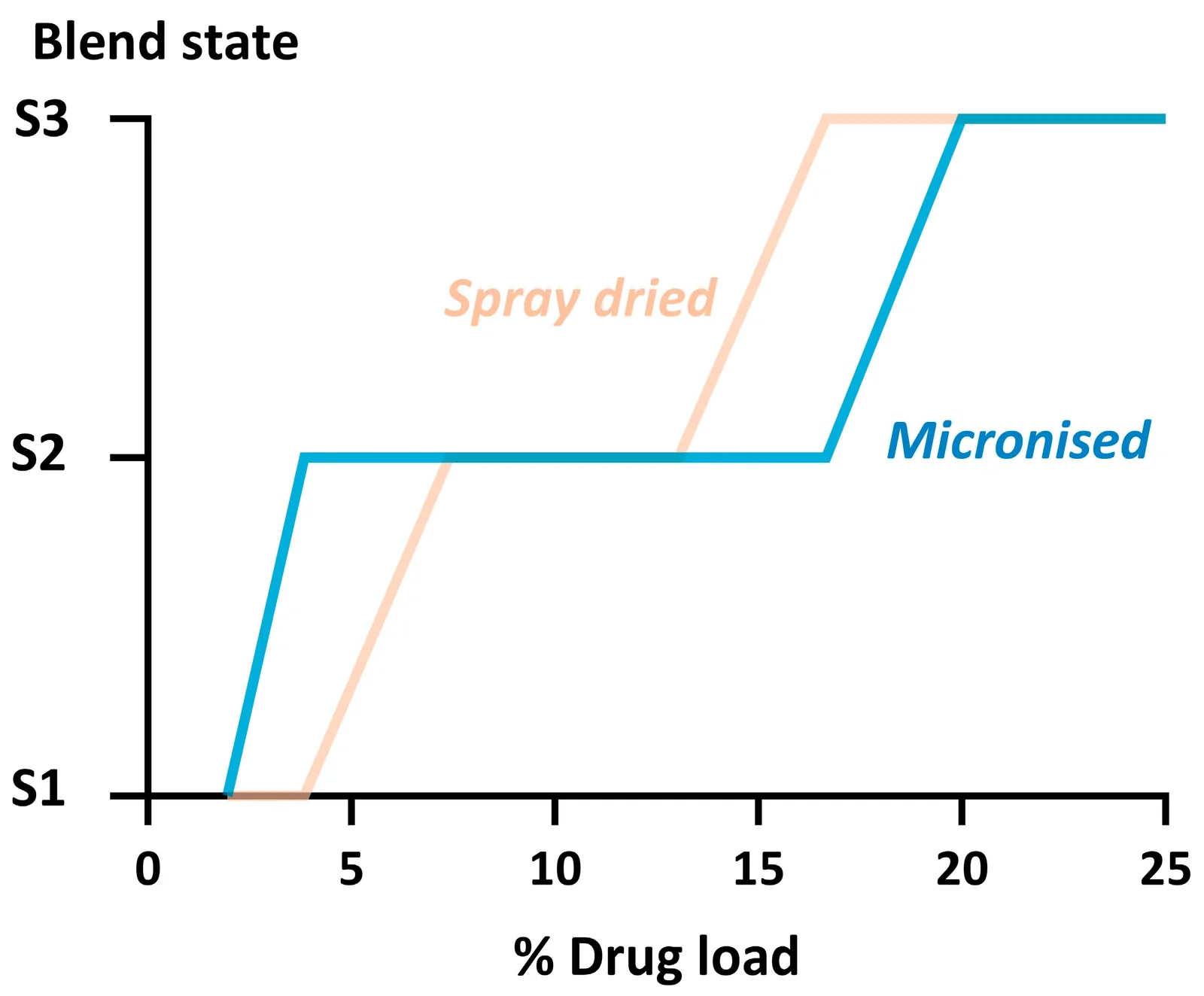
Evolution of blend states for adhesive mixtures of micronized and spray-dried drugs in the form of a blend state map.

For these mixtures, two carrier saturation levels were thus identified: the first when the carrier irregularities were filled with fine drug particles (end of blend state 1), and the second when the thickest possible adhesive layer was formed (end of blend state 2). In Figure 17, the differences in evolution of blend state with drug concentration between micronized and spray-dried particles are illustrated as a blend state map.

It is evident that the main factor controlling the observed differences in blend state and blend mechanical properties between the mixtures was whether a crystalline or an amorphous form of the drug was used. It is thus proposed that the significant properties of the drug particles influencing the evolution of blend architecture were the particle shape and deformability.

### 4.3. Effect of Drug Load on the Aerosolization Propensity of Adhesive Mixtures

The degree of dispersion of the mixtures during aerosolization, as assessed using the *FPD* and the *FPF*, §were generally somewhat higher for the Monodose device than for the Screenhaler device. Moreover, there were differences in the degree of dispersion depending on the mixture type for both inhalers, indicating that the more intense processing in the Monodose inhaler did not eliminate the importance of mixture structure during aerosolization. Thus, the degree of blend dispersion was determined by a combination of the blend structure and the mechanism of action of the inhaler.

For the Screenhaler, at low drug concentrations (1.96% drug), the spray-dried particles gave a very low *FPF,* markedly lower than that of the mixtures containing micronized particles. Since the spray-dried particles packed more densely into carrier surface cavities (blend state 1), they became more difficult to detach. For the Monodose device, the *FPF* was higher for the mixtures at the lowest drug concentration, which can be explained by more intense collisions between adhesive units and with the capsule and inhaler walls during emptying of the device, facilitating detachment by an inertia-driven mechanism.

As the drug load increased to 7.41%, both the *FPD* and *FPF* increased for both devices. At this concentration, a significant portion of the drug particles was adsorbed onto the enveloped carrier surfaces (blend state 2), and the particles were more easily detached from this position than from the surface cavities. However, for the respective drug, the *FPF* remained lower for mixtures containing spray-dried particles than for micronized particles due to the different distribution of particles between blend states 1 and 2 (Figure 17). For the two highest drug loads (13.0 and 16.7% *w*/*w*), the *FPD* leveled out for the Screenhaler but continued to increase for the Monodose inhaler. The *FPF* also tended to level out at these drug loads, but not for all mixtures. At these high drug loads, when the drug layer became thicker and more heterogeneous, and self-agglomerates started to form in the mixture (i.e., blend state S2b and gradual transition to S3), the spray-dried particles thus dispersed more easily than the micronized particles. This is consistent with the discussion above that spray-dried particles form a more voluminous adhesive layer of lower strength on the enveloped carrier surface than micronized particles and thus can be more easily detached.

Generally, the *FPF*-drug load relationships were complicated, and their progression was dependent on both the properties of the drug particles and the type of inhaler. The *FPF*-drug load relationships were more closely gathered for the Monodose device, and for *TS,* overlapping relationships were observed. The Monodose inhaler clearly had a higher dispersion capacity than the Screenhaler in this study, i.e., it was more effective at detaching and de-agglomerating drug particles.

To summarize, when drug particles are prone to pack densely within carrier surface cavities (in blend state 1), the bonds formed between drug particles are strong with high resistance toward detachment, resulting in a low *FPF*. At intermediate drug loads, this can also result in a relatively low *FPF* due to an unfavorable distribution of drug particles, i.e., a large proportion of drug particles are located in surface cavities with low detachment propensity. Finally, when the multiple particle adhesive layer (blend state 2b) is voluminous, and thus probably of low strength, detachment is facilitated, and the *FPF* continues to increase. At high drug loads, the dispersibility of drug self-agglomerates may also influence aerosolization and *FPF*.

The *MMAD* were generally low and similar to the physical particle diameters (Table 2). Consequently, the drug particles dispersed in the NGI were primary particles and micro-agglomerates with a low agglomeration number. Mixtures containing spray-dried materials generally exhibited higher *MMAD* values than the micronized ones, indicating that a larger proportion of micro-agglomerates impacted in the NGI. Overall, mixtures of spray-dried *SS* exhibited the lowest *FPF* and highest *MMAD,* indicating that these mixtures were the most difficult to disperse.

### 4.4. Correlations Between Mechanical and Aerosolization Properties

It was earlier reported [19] that the progression of *FPF* with drug load for a series of adhesive mixtures of budesonide and a lactose carrier was similar to the corresponding progression of some shearing parameters. Thus, dispersion and shearing had a similar dependency on the blend structure. To substantiate this conclusion, the mixtures used in this study were also subjected to a correlation test (Figure 15). Since only four selected drug concentrations were used in the impactor experiments, observed associations between mechanical and dispersion properties should be interpreted as trends and not as significant correlations. For micronized *SS*, no significant correlations were observed, while for the other drug materials, the correlations between *FPF* and indications of packing density and flowability support the notion that the architecture of an adhesive mixture is a critical factor governing the overall performance of adhesive mixtures, including packing density, flowability, and aerosolization behavior. Thus, adhesive mixtures may be characterized by parallel structure–property relationships.

The correlations between mechanical properties, especially shearing parameters, and *FPF* (Table A1 and Table A2) indicate that both shearing and dispersion are dictated by the friction resistance between adhesive units. In an earlier study [17], it was shown that the adhesion units impacted in the pre-separator during a NGI experiment had an appearance similar to the starting adhesion units, and it was proposed that the dominating mechanism of dispersion of the drug fines was surface erosion. The correlation between shearing and dispersion observed in this study is also consistent with the conception of erosion as the main dispersion mechanism. This mechanism may, however, be restricted to adhesive mixtures in blend state 2, while in blend state 1, where the fine particles are shielded in cavities, dispersion by detachment due to inertia during collisions seems most reasonable.

**Figure 18:**
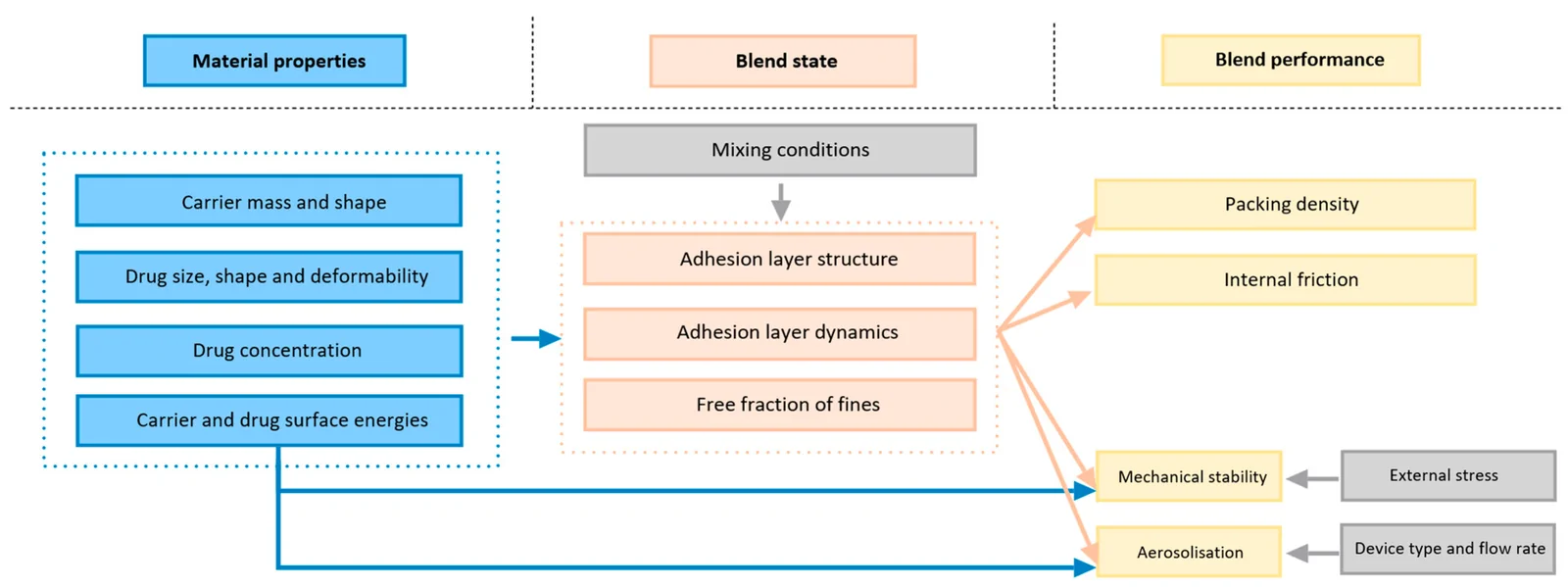
Flowchart summarizing, as a consecutive relationship, the material properties controlling the structure of the adhesive mixture and the subsequent blend structure–property relationships (blend performance).

Based on this and earlier research described in the Section 1, a proposed consecutive relationship between material properties, blend state, and blend properties (blend performance) for the type of binary adhesive mixtures used in this study is presented in Figure 18.

## 5. Conclusions

In this paper, the structure and properties of adhesive mixtures with drugs of different solid forms have been investigated, and the key findings are:

1. For both drug particle types, the overall evolution of the mixture structure with increasing drug load was consistent with the blend state concept.
2. The drug concentrations at which blend state transitions occurred were influenced by the properties of the drug particles. Spray-dried drug particles were, firstly, more prone to position into grooves and valleys of the carrier particles; secondly, they formed a more voluminous adhesive layer on the enveloped carrier surfaces; and, thirdly, they formed self-agglomerates at lower drug loads.
3. The type of drug particles influenced the mechanical and aerosolization properties of the adhesive mixtures and thus a series of blend structure—blend property links applied to the mixtures. The structure-aerosolization relationship applied to both types of inhalers studied, although the inhaler producing generally higher *FPD* and *FPF* tended to better accommodate the effect of drug particle type.
4. The correlation between shearing and dispersion observed substantiates that erosion is a dominating dispersion mechanism of adhesive units in blend state 2.
5. The critical particle properties causing the observed performance differences between spray-dried and micronized particles are proposed to be their shape and deformability.

In the systems investigated here, changes in physical particle properties influenced both blend structure and blend properties, consistent with the proposed structure-performance link. Control of drug particle properties, for example, by using spray-dried materials, may be useful in the optimization of inhalation powders and maximizing drug load. However, the *FPF* of crystalline and spray-dried drugs used in this study did not follow a consistent difference over the whole range of drug concentrations, and a general statement on which solid form of drug shows a superior degree of aerosolization can consequently not be given. The spray-dried drugs showed lower *FPF* than the crystalline drugs at low drug loads and similar or higher at high drug loads, i.e., showed lower propensity to aerosolize while located in blend state 1 (active sites) and higher propensity while located in blend state 2. The difference in aerosolization propensity dependent on the localization of the drug on the carrier surface can be explained by a different packing density of the drug particles at the respective site, i.e., higher packing density and layer strength in carrier depressions but lower at the enveloped surfaces due to the formation of a more voluminous adhesion layer. The choice of solid form of drug depends therefore on the drug concentration and the morphology of the carrier. This raises the question of the optimal drug concentration in an adhesive mixture for a given drug dose. For a given drug dose, the required weight of the adhesive mixture will vary with drug concentration in the mixture. It should be an interesting step forward to also compare the aerosolization of such a series of adhesive mixtures with a consistent dose but of varying mixture weights in order to better understand the ideal blend state. In addition, in order to further improve the understanding of the role of blend architecture for the clinical effect of an inhalation powder, in vivo studies need to be conducted.

The use of spray-dried particles in powder inhalation products may be limited by the inherent lack of stability of amorphous materials, an issue that requires careful consideration in product development. Assessment of long-term storage stability and large-scale manufacturing was outside the scope of the present study.

The findings further highlight that a proper choice of methods in a test protocol for mechanical analysis of adhesive mixtures is essential to understand mixture structure and to predict mixture performance. In this context, the angle of internal friction may represent an interesting approach to understand the development of mixture structure with increasing drug load.

## Figures and Tables

**Table 1:** Weights and weight proportions of drug in the binary mixtures.

Weight of Drug (g)	Weight Proportion of Drug (%)
1.0	1.96
2.0	3.85
4.0	7.41
6.0	10.7
7.5	13.0
10.0	16.7
12.5	20.0

**Table 2:** Particle properties of starting materials. Mean values and relative standard deviations in % within brackets. (n = 3).

Material	*ρ*_p_ (g/cm^3^)	*D*10 (µm)	*D*50 (µm)	*D*90 (µm)	Span (−)	*ρ*_b_ (g/cm^3^)	*HR* (−)
Lactopress	1.54 (0.0553)	45.2 (3.69)	103 (1.98)	192 (1.57)	1.42	0.643 (0.162)	1.12 (0.588)
*TS*_m_	1.34 (0.541)	0.359 (1.39)	1.94 (1.54)	4.13 (1.06)	1.94	0.0924 (3.18)	3.74 (4.01)
*TS*_sd_	1.30 (0.0154)	0.357 (1.18)	1.89 (0.529)	4.10 (1.90)	1.98	0.299 (3.51)	1.67 (4.19)
*SS*_m_	1.33 (0.00754)	1.05 (1.62)	2.39 (1.25)	4.74 (0.570)	1.54	0.138 (4.40)	1.38 (0.724)
*SS*_sd_	1.28 (0.164)	1.02 (0.588)	2.11 (0.171)	4.03 (1.29)	1.43	0.309 (2.90)	1.67 (0.479)

*ρ*_p_ = Particle gas pycnometry density; *D* _=_ Physical diameter of particles at 10, 50 and 90% of cumulative undersize distribution; Span = Span of particle size distribution; *ρ*_b =_ Bulk density; *HR* _=_ Hausner ratio; *TS*_m_ = Micronized terbutaline sulfate; *TS*_sd_ = Spray-dried terbutaline sulfate; *SS*_m_ = Micronized salbutamol sulfate; *SS*_sd_ = Spray-dried salbutamol sulfate.

**Table 3:** Shearing properties of starting materials. Mean values and relative standard deviations in % within brackets. (n = 3).

Material	*τ*_0_ (kPa)	*ff*_c_ (%)	*φ* (^o^)
Lactopress	0.13 (7.7)	10.0 (7.48)	29.1 (3.50)
*TS*_m_	0.82 (12)	2.09 (9.56)	48.6 (10.3)
*TS*_sd_	1.21 (21.4)	1.47 (10.9)	41.7 (10.3)
*SS*_m_	0.60 (17)	2.75 (9.81)	42.0 (8.25)
*SS*_sd_	1.37 (18.9)	1.42 (9.15)	35.6 (13.5)

*τ*_0_ = Cohesion strength; *ff*_c_ = Flow function coefficient; *φ =* Angle of internal friction; *TS*_m_ = Micronized terbutaline sulfate; *TS*_sd_ = Spray-dried terbutaline sulfate; *SS*_m_ = Micronized salbutamol sulfate; *SS*_sd_ = Spray-dried salbutamol sulfate.

## Data Availability

The research data generated during the study is available on request.

## Abbreviations

The following abbreviations are used in this paper:

*D*	Physical diameter of particles (m)
*ED*	Emitted dose (g)
*ff*_c_	Flow function coefficient (flow index) (−)
*FPD*	Fine particle dose (g)
*FPF*	Fine particle fraction (%)
*HR*	Hausner ratio (−)
*k*	Unconsolidated powder permeability (m^4^/Ns)
*MMAD*	Mass median aerodynamic diameter of particles (m)
*NGI*	Next generation impactor
Δ*P*	Pressure drop (N/m^2^)
*RH*	Relative humidity (%)
*SS*	Salbutamol sulfate
*TS*	Terbutaline sulfate
*ρ*_b_	Unconsolidated powder bulk density (kg/m^3^)
*ρ*_p_	Particle gas pycnometry density (kg/m^3^)
*τ*_0_	Cohesion strength (N/m^2^)
*φ*	Angle of internal friction (^o^)

## Appendix A

**Table A1:** Correlations (*p* < 0.1) between fine particle fraction (*FPF*) and mechanical properties (divided into four classes) for adhesive mixtures with different drugs used in reference [19] and in this study.

Class	Correlations for Micronized Budesonide ^1^	Correlations for Spray- Dried Terbutaline Sulfate ^2^	Correlations for Micronized Terbutaline Sulfate ^2^	Correlations for Spray-Dried Salbutamol Sulfate ^2^	Correlations for Micronized Salbutamol Sulfate ^2^
Unsettled bulk density	No	Yes	No	No	No
Compression flow	No	No	Yes	No	No
Shear flow ^3^	Yes	Yes	Yes	Yes	No
Aeration test	No	No	No	No	No

^1^ Results from reference [19]. ^2^ Results from this study. ^3^ Shear flow parameters for which correlations were obtained varied between the mixtures (see Table A2).

**Table A2:** Correlations (*p* < 0.1) between fine particle fraction (*FPF*) and the different shear parameters for adhesive mixtures with different drugs used in reference [19] and in this study.

Class	Correlations for Micronized Budesonide ^1^	Correlations for Spray-Dried Terbutaline Sulfate ^2^	Correlations for Micronized Terbutaline Sulfate ^2^	Correlations for Spray-Dried Salbutamol Sulfate ^2^	Correlations for Micronized Salbutamol Sulfate ^2^
Unconfined yield strength	Yes	No	No	No	No
Flow function coefficient	No	Yes	Yes	Yes	No
Angle of internal friction	No	No	Yes	No	No
Cohesion strength	No	Yes	No	Yes	No

^1^ Results from reference [19]. ^2^ Results from this study.
